# Systematic error detection in experimental high-throughput screening

**DOI:** 10.1186/1471-2105-12-25

**Published:** 2011-01-19

**Authors:** Plamen Dragiev, Robert Nadon, Vladimir Makarenkov

**Affiliations:** 1 Département d'informatique, Université du Québec à Montréal, C.P. 8888 succ. Centre-Ville, Montreal (QC) H3C 3P8, Canada; 2 Department of Human Genetics, McGill University, 1205 Dr. Penfield Ave. Montreal, QC, H3A 1B1; 3 McGill University and Genome Quebec Innovation Centre, 740 Dr. Penfield Ave., Montreal, QC, H3A 1A4, Canada

## Abstract

**Background:**

High-throughput screening (HTS) is a key part of the drug discovery process during which thousands of chemical compounds are screened and their activity levels measured in order to identify potential drug candidates (i.e., hits). Many technical, procedural or environmental factors can cause systematic measurement error or inequalities in the conditions in which the measurements are taken. Such systematic error has the potential to critically affect the hit selection process. Several error correction methods and software have been developed to address this issue in the context of experimental HTS [1-7]. Despite their power to reduce the impact of systematic error when applied to error perturbed datasets, those methods also have one disadvantage - they introduce a bias when applied to data not containing any systematic error [6]. Hence, we need first to assess the presence of systematic error in a given HTS assay and then carry out systematic error correction method if and only if the presence of systematic error has been confirmed by statistical tests.

**Results:**

We tested three statistical procedures to assess the presence of systematic error in experimental HTS data, including the χ^2 ^goodness-of-fit test, Student's t-test and Kolmogorov-Smirnov test [8] preceded by the Discrete Fourier Transform (DFT) method [9]. We applied these procedures to raw HTS measurements, first, and to estimated hit distribution surfaces, second. The three competing tests were applied to analyse simulated datasets containing different types of systematic error, and to a real HTS dataset. Their accuracy was compared under various error conditions.

**Conclusions:**

A successful assessment of the presence of systematic error in experimental HTS assays is possible when the appropriate statistical methodology is used. Namely, the t-test should be carried out by researchers to determine whether systematic error is present in their HTS data prior to applying any error correction method. This important step can significantly improve the quality of selected hits.

## Background

High-throughput screening (HTS) is a modern technology used by drug researchers to identify pharmacologically active compounds [10]. HTS is a highly automated early-stage mass screening process. Contemporary HTS equipment allows for testing more than 100,000 compounds a day. HTS serves as a starting point for rapid identification of primary hits that are then further screened and evaluated to determine their activity, specificity, and physiological and toxicological properties [2]. As a highly sensitive test system, HTS requires both precise measurement tools and dependable quality control. The absence of standardized data validation and quality assurance procedures is recognised as one of the major hurdles in modern experimental HTS [11-13]. Acknowledging the importance of automatic quality assessment and data correction systems, many researchers have offered methods for eliminating experimental systematic artefacts which, if left uncorrected, can obscure important biological or chemical properties of screened compounds (false negatives) and can seemingly indicate biological activity when there is none (false positives) [1-7,10-16].

Systematic error may be caused by various factors, including robotic failures and reader effects, pipette malfunction or other liquid handling anomalies, unintended differences in compound concentrations due to agent evaporation or variation in the incubation time and temperature differences, and lighting or air flow present over the course of the entire screen [2,6]. Unlike random error that produces measurement noise and usually has minimal impact on the whole process, systematic error produces measurements that are systematically over- or underestimated. Systematic error may be time dependent, introducing biases in individual plates or subsets of consecutive plates, but it may also affect an entire HTS assay (i.e., all screened plates). In practice, systematic error is almost always location related. The under- or overestimation affects compounds located in the same row or column or in the same well location across the screened plates. The row and column effects may be persistent across the assay affecting repeatedly the same rows and columns on different plates or may vary from plate to plate, perturbing some rows and columns within a particular plate only [6]. Plate controls are used in HTS to ensure the accuracy of the activity measurements being taken. Controls are substances with stable well-known activity levels. They might be positive (i.e., a strong activity effect is observed) or negative (i.e., no any activity effect is observed). Controls help to detect plate-to-plate variability and determine the level of background noise.

The following normalisation and pre-processing methods have been widely used in experimental HTS to remove plate-to-plate variation and make plate measurements comparable across plates [6,13]:

• *Percent of control - *the following formula is used:

${\hat{x}}_{ij}=\frac{x_{ij}}{\mu_{pos}}$, where *x_ij _*is the raw measurement of the compound in well (*i, j*), ${\hat{x}}_{ij}$ is the normalized value of *x_ij_*, and *μ_pos _*is the mean of positive controls.

• *Control normalization *(known also as *normalized percent inhibition transformation*) is based on the following formula:

${\hat{x}}_{ij}=\frac{x_{ij}-\mu_{neg}}{\mu_{pos}-\mu_{neg}}$, where *x_ij _*is the raw measurement of the compound in well (*i*, *j*), ${\hat{x}}_{ij}$ is the normalized value of *x_ij_*, *μ_pos _*is the mean of positive controls, and *μ_neg _*is the mean of negative controls.

• *Z-score *normalization is carried out as follows:

${\hat{x}}_{ij}=\frac{x_{ij}-\mu }{\sigma }$, where *x_ij _*is the raw measurement of the compound in well (*i, j*), ${\hat{x}}_{ij}$ is the normalized value of *x_ij_*, *μ *is the mean of all the measurements of the given plate, and *σ *is the standard deviation of all the measurements of the given plate.

• *B-score *(i.e., *Best score *normalization [3]) is carried out as follows:

First, a two-way median polish procedure [17] is performed to account for row and column effects of the plate. The resulting residuals within each plate are then divided by their median absolute deviation, *MAD*. It is worth noting that there is an additional smoothing step that could be applied across plates (see the original article [3] for a description of the smoothing). This optional smoothing step was not applied however in [[5,6] and [17]].

The residual *(r_ijp_) *of the measurement in row *i *and column *j *on the *p*^th ^plate is obtained as follows by a two-way median polish procedure (Equation 1):

(1) $$r_{ijp}=x_{ijp}-{\hat{x}}_{ijp}=x_{ijp}-\left({\hat{\mu }}_{p}+{\hat{R}}_{ip}+{\hat{C}}_{jp}\right).$$

The residual is defined as the difference between the observed result (*x_ijp_*) and the fitted value ${\hat{x}}_{ijp}$, defined as the estimated average of the plate (${\hat{\mu }}_{p}$) + estimated systematic measurement offset $\left({\hat{R}}_{ip}\right)$ for row *i *of plate *p *+ estimated systematic measurement column offset $\left({\hat{C}}_{jp}\right)$ for column *j *of plate *p*. For each plate *p*, the adjusted median absolute deviation (*MAD_p_*) is then obtained from the *r_ijp_*'s.

*Median absolute deviation *(*MAD*)*- *a robust estimate of spread of the *r_ijp_*'s values is computed as follows: $median\left\{\;\left|\;r_{ijp}-median\left(r_{ijp}\right)\;\right|\;\right\}$.

The B-score normalized measurements are then calculated as follows:

(2) $$B-score=\frac{r_{ijp}}{MAD_{p}}.$$

The B-score normalization was introduced by a team of *Merck Frosst *researchers [3] as a systematic error correction method.

• *Well correction *is another advanced systematic error correction technique [5,6] used to remove systematic biases affecting the assay's wells, rows or columns, and spread across all the plates of the assay. It consists of two main steps:

1. Least-squares approximation of the data carried out separately for each well location of the assay;

2. Z-score normalization of the data within each well location of the assay (i.e., the Z-score normalization is performed across all the plates of the assay).

In the HTS workflow, the normalization/data correction phase is usually followed by the *hit selection *process. During this process the most active compounds are identified as *hits *and selected for additional screens. A predefined threshold is usually established to select hits [13]. Depending on the specifics of the research study, one may be looking for compounds whose activity level is greater than the defined threshold (i.e., activation assay) or interest may lie in the compounds whose measurements are below the defined threshold (i.e., inhibition assay). In this study, we always assume the latter case where the hits are the compounds with the smallest measurement values. The threshold for defining hits is usually expressed using the mean value and standard deviation of the considered measurements. The most widely used threshold is *μ-*3*σ*, where *μ *is the mean value and *σ *is the standard deviation of the considered measurements. Hits can be selected globally, over the whole assay, when the mean and standard deviation of all assay compounds are calculated, or on a plate-by-plate basis, when the mean and standard deviation of the compounds of each single plate are considered [6,13].

**Figure 1:**
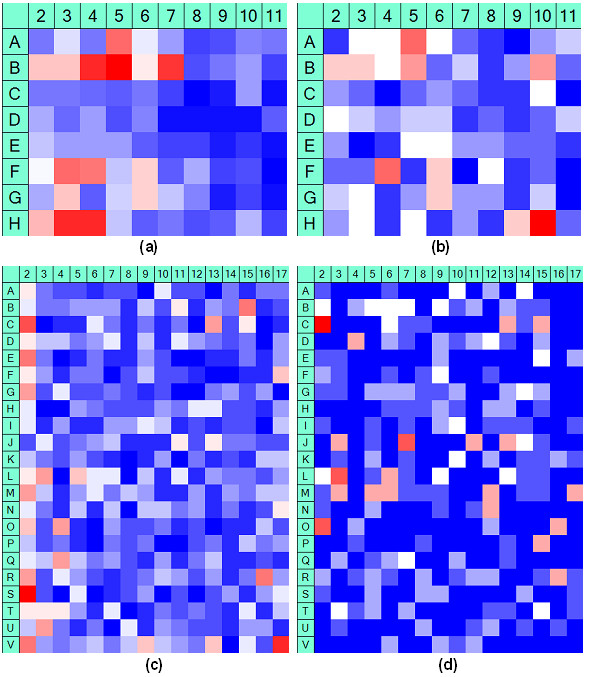
**Systematic error in experimental HTS data**. Hit distribution surfaces for the McMaster (cases (a) and (b) - 1250 plates - [18]) and Princeton (cases (c) and (d) - 164 plates - [19]) Universities experimental HTS assays. Values deviating from the plate means for more than 2 standard deviations *- *cases (a) and (c), and for more than 3 standard deviations - cases (b) and (d) were selected as hits. The well, row and column positional effects are shown (the wells containing controls are not presented).

The presence of systematic error in a HTS assay can be identified and visualized using its *hit distribution surface *[4,6]. Such a surface can be computed by determining the number of selected hits for each well location. In the ideal case when systematic error is absent, we expect that the hits are evenly distributed over the well locations. However, this expectation is not always fulfilled in real datasets (see Figure 1). This figure presents the hit distribution surfaces computed for two hit selection thresholds,*μ-*2*σ *and *μ-*3*σ*, of two experimental HTS screens performed at McMaster (Figure 1a,b - [18]) and Princeton (Figure 1c,d - [19]) Universities. The row and column effects in the hit distributions across plates are easily noticeable here, especially in the case of a lower (i.e., *μ-*2*σ*) hit selection threshold. The dataset provided by the Chemistry Department of Princeton University consists of a screen of compounds that inhibit the glycosyltransferase MurG function of *E. coli *[19]. The experimental data for 164 plates were considered. According to the ChemBank description, this assay has been obtained during a screen that measured the binding of MurG to a fluorescent (fluorescein-labelled) analogue of UDP-GlcNAc. Positives were defined as compounds that inhibit binding of GlcNAc to MurG. The McMaster assay was originally used as a benchmark in *McMaster Data Mining and Docking Competition *[18]. The McMaster dataset, which will be examined in detail in this study, consists of compounds intended to inhibit the *E. coli Dihydrofolate reductase *(DHFR). The screen of 50,000 training molecules selected by the organizers of McMaster Competition yielded 96 primary hits, then, 12 potent hits (i.e., hits confirmed by dose response analysis), the majority of which were novel DHFR inhibitors that fell into 3 broad structural classes [18].

**Figure 2:**
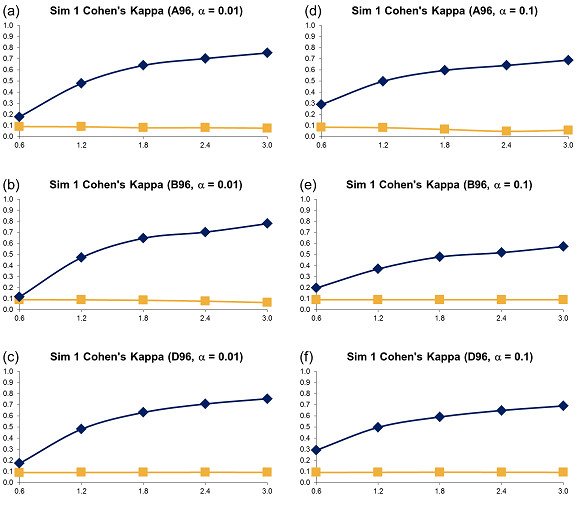
**Simulation 1, Plate Size: 96 wells - Cohen's Kappa vs Error Size**. Systematic error size: 10% (at most 2 columns and 2 rows affected). First column: (a) - (c): *α *= 0.01; Second column: (d) - (f): *α *= 0.1. Systematic Error Detection Tests: (◆) t-test and (■) K-S test.

It is worth noting that the application of sophisticated pre-processing HTS techniques does not always guarantee data improvement. Moreover, the application of systematic error correction methods on error-free HTS assays will produce data in which certain activity measurements will be biased [6]. The result of such a misuse of data pre-processing methods can lead to a dramatically inaccurate hit selection. Makarenkov et al. (see Figure 2 and Figure 4, cases a and c, in [6]) showed that all data correction methods introduce a bias when applied to error-free HTS data. This bias can be less important (e.g., in the case of the Well correction procedure) or very significant (e.g., in the case of the B-score method). Hence, the data correction methods should be applied with caution and only in situations when the presence of systematic error in the given assay has been demonstrated by an appropriate statistical methodology. Assessing the presence of systematic error in experimental HTS is the main focus of this article.

**Figure 3:**
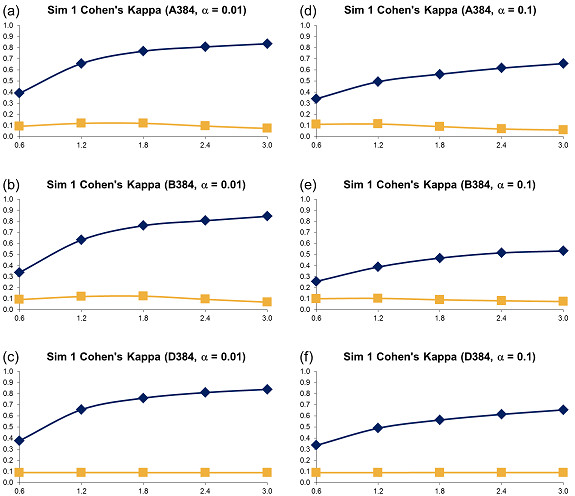
**Simulation 1, Plate Size: 384 wells - Cohen's Kappa vs Error Size**. Systematic error size: 10% (at most 4 columns and 4 rows affected). First column: (a) - (c): *α *= 0.01; Second column: (d) - (f): *α *= 0.1. Systematic Error Detection Tests: (◆) t-test and (■) K-S test.

**Figure 4:**
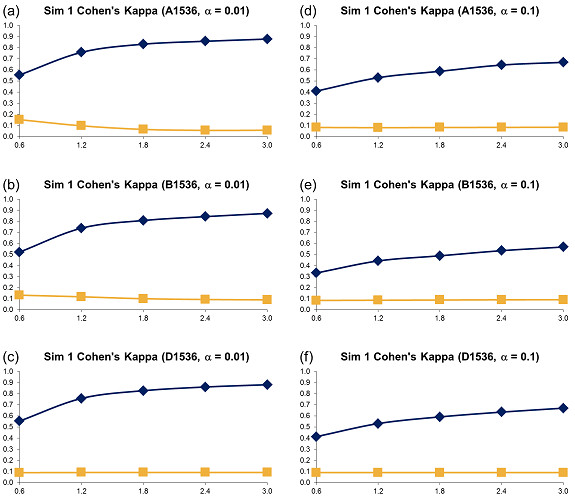
**Simulation 1, Plate Size: 1536 wells - Cohen's Kappa vs Error Size**. Systematic error size: 10% (at most 8 columns and 8 rows affected). First column: (a) - (c): *α *= 0.01; Second column: (d) - (f): *α *= 0.1. Systematic Error Detection Tests: (◆) t-test and (■) K-S test.

## Methods

### Data description

In this study we consider an experimental assay provided by the HTS laboratory of McMaster University. This assay was called *Test assay *and used as a benchmark in McMaster Data Mining and Docking Competition [18]. McMaster *Test assay *consists of 50,000 different chemical compounds whose potential to inhibit the *E. coli *DHFR was tested. Each of the 50,000 considered compounds was screened in duplicate; two copies of each of the 625 plates were run through the HTS equipment; 1250 plates in total, with wells arranged in 8 rows and 12 columns, were screened; columns 1 and 12 of each plate were used for positive and negative controls and were, therefore, not considered in our study. Thus, every plate comprised 80 different compounds. The exact experimental conditions of *Test assay *are reported in [18]. The competition organizers defined as *primary hits *the compounds that reduced the DHFR of *E. coli *to 75% of the average residual activity of the high controls. Two lists of hits were published (for more details, the reader is referred to: http://www.info2.uqam.ca/~makarenv/experimental_actives.pdf). The first list, called a *consensus hits list*, contained all compounds that were classified as hits in both of their replicate measurements (i.e., both measurement values were lower than or equal to 75% of the reference controls). Only 42 of all the 50,000 tested compounds were declared consensus hits. The second list, called an *average hits list*, contained 96 compounds classified as hits when the average value of the two HTS measurements was lower than or equal to 75% of the reference controls. Obviously, all consensus hits were also average hits. A secondary screening of the 96 average hits was also performed in order to determine their activity in different concentrations. As result of the secondary screening, 12 of the average hits were identified as *D-R hits *(i.e., hits having *well-behaved dose-response curves*).

### Generating systematic error

We simulated data in order to evaluate the performances of the systematic error detection tests. First, we generated error-free datasets consisting of random normally distributed data. The basic data format adopted here was that of the McMaster dataset - 1250 plates, each containing 96 wells arranged in 8 rows and 12 columns. In addition, we also generated two other basic datasets which were 4 and 16 times bigger. They also included 1250 plates, each of them comprising 384 (16 × 24) and 1536 (32 × 48) wells, respectively. It is worth noting that 96, 384 and 1536-well plates are the most typical plate formats used in the modern HTS.

An assay was defined as an ordered set of plates *PL_p_*, where *p *(1 ≤ *p *≤ 1250) is the plate number. Each plate, *PL_p_*, can be viewed as a matrix of experimental HTS measurements *x_ijp_*, where *i *(1 ≤ *i *≤ *N_R_*) is the row number, *j *(1 ≤ *j *≤ *N_C_*) is the column number, and *N_R _*and *N_C _*are, respectively, the number of rows and columns in *PL_P_*. The generated values *X_ijp_*'s followed the standard normal distribution ~N(0, 1).

Then, the hits were added to the datasets. Several hit percentages, *h*, were tested in our simulations: *h *= 0.5, 1, 2, 3, 4 and 5%. The locations and values of hits were chosen randomly. The probability of each well in each plate to contain a hit was *h*%. The values of hits followed a normal distribution with the parameters ~N(*μ *- 5*SD*, *SD*), where *μ *and *SD *are the mean value and standard deviation of the error-free dataset.

In total, five types of datasets, presented in Table 1, containing different kinds of systematic and/or random error were generated and tested.

**Table 1:** Five types of HTS datasets containing different kinds of systematic and/or random error generated and tested in this study

Error type	Generation of error-affected measurements
**A**. Datasets with both *column and row systematic errors *which are constant across all assay plates.	${x^{\prime }}_{ijp}=x_{ijp}+r_{i}+c_{j}+Rand_{ijp}$, 1 ≤ *i *≤ 8, 1 ≤ *j *≤ 12, 1 ≤ *p *≤ 1250.

**B**. Datasets with the *column systematic error only *which is constant across all plates.	${x^{\prime }}_{ijp}=x_{ijp}+c_{j}+Rand_{ijp}$, 1 ≤ *i *≤ 8, 1 ≤ *j *≤ 12, 1 ≤ *p *≤ 1250.

**C**. Datasets with the *well systematic error *which is constant across all plates.	${x^{\prime }}_{ijp}=x_{ijp}+w_{ij}+Rand_{ijp}$, 1 ≤ *i *≤ 8, 1 ≤ *j *≤ 12, 1 ≤ *p *≤ 1250.

**D**. Datasets with the *variable column and row systematic error *which are different for each plate.	${x^{\prime }}_{ijp}=x_{ijp}+r_{ip}+c_{jp}+Rand_{ijp}$, 1 ≤ *i *≤ 8, 1 ≤ *j *≤ 12, 1 ≤ *p *≤ 1250.

**E**. Datasets with the *random error only *(i.e., systematic error was absent).	${x^{\prime }}_{ijp}=x_{ijp}+Rand_{ijp}$, 1 ≤ *i *≤ 8, 1 ≤ *j *≤ 12, 1 ≤ *p *≤ 1250.

where: ${x^{\prime }}_{ijp}$ is the error-affected value in well *i,j *(row *i*, column *j*) of plate *p*.

*x_ijp _*is the original value in well *i,j *of plate *p *in the error-free dataset.

*r_i _*is the systematic error in row *i *(constant over all plates); it had a normal distribution with the parameters ~N(0, *C*).

*x_j _*is the systematic error in column *j *(constant over all plates); it had a normal distribution with the parameters ~N(0, *C*).

*w_ij _*is the systematic error that affects well *i,j *(row *i*, column *j*) and is the same for all plates; it had a normal distribution with the parameters ~N(0, *C*).

*r_ip _*is the systematic error in row *i *of plate *p*; it had a normal distribution with the parameters ~N(0, *C*).

*c_jp _*is the systematic error in column *i *of plate *p*; it had a normal distribution with the parameters ~N(0, *C*).

*Rand_ijp _*is the random error affecting well *i,j *(row *i*, column *j*) of plate *p*; it had a normal distribution with the parameters ~N(0, 0.3×*SD*).

Datasets for *C *= 0, 0.6×*SD*, 1.2×*SD*, 1.8×*SD*, 2.4×*SD *and 3×*SD *were generated and tested, where *μ *is the mean and *SD *is the standard deviation of the error-free dataset.

In order to render our simulation study more realistic, we limited the number of rows, columns and wells affected by systematic error. Typically, in real HTS assays only some of the error parameters (i.e., *r_i_*, *c_j_*, *w_ij_*,*r_ip _*and *c_jp_*, see Table 1) are non null and only a few columns and rows are biased by systematic error. In datasets of types A and B, the number of rows and columns affected by systematic error as well as their locations were chosen randomly. These parameters were identical for all the plates of the assay. In datasets of type D, the number of rows and columns affected by systematic error as well as their locations were also randomly selected, but these parameters were different for different plates of the assay. In datasets of type C, the number of biased wells and their locations were randomly selected and were the same for all assay plates. The datasets used in our simulations were subject to the following constraints. For the 96-well plates, at most 2 rows and 2 columns (cases A, B and D), and not more than 10% of the wells (case C) were affected by systematic error. For the 384-well plates, the limits were 4 rows, 4 columns and 10% of the wells, whereas for the 1536-well plates, systematic error affected at most 8 rows, 8 columns and 10% of wells.

### Systematic error detection tests

Three systematic error detection methods, including the t-test, the *χ^2 ^*goodness-of-fit test and Discrete Fournier Transform procedure followed by the Kolmogorov-Smirnov test, were examined in this study in the context of experimental HTS.

#### t-test

The first systematic error detection test was based on the classical two-sample Student's t-test for the case of samples with different sizes. In Simulation 1, we carried out this test on every row and every column of each assay. In Simulation 2, we applied it to the rows and columns of the assay's hit distribution surfaces. In both cases, we divided the data into two independent subsets (i.e., samples). The first subset contained the measurements of the tested row or column while the second subset consisted of all remaining plate measurements. In this test, the null hypothesis *H_0_*, was that the selected row or column does not contain systematic error. If systematic error is absent, then the mean of the given row or column is expected to be close to the mean of the rest of the data in the given plate or hit distribution surface. For the two samples in hand: *S_1 _*with *N_1 _*elements and *S_2 _*with *N_1 _*elements, we first calculated the two sample variances $s_{1}^{2}$ and $s_{2}^{2}$, and then their weighted average (Equation 3):

(3) $$s_{p}^{2}=\frac{\left(N_{1}-1\right)\times s_{1}^{2}+\left(N_{2}-1\right)\times s_{2}^{2}}{N_{1}+N_{2}-2}.$$

The value of the *t-statistic *was then obtained as presented in Equation 4:

(4) $$t=\frac{\mu_{1}-\mu_{2}}{\sqrt{s_{p}^{1}\left(\frac{1}{N_{1}}+\frac{1}{N_{2}}\right)}},$$

where *μ_1 _*is the mean of the sample *S_1 _*and *μ_2 _*is the mean of the sample *S_2_*. The calculated *t-statistic *was then compared to the corresponding critical value for the chosen statistical significance level *α *(the *α *values equal to 0.01 and 0.1 were used in our simulations) in order to decide whether or not *H_0 _*should be rejected. While assuming homogeneity of variance in the construction of the t-test, the computation can be optimized using the equivalent contrasts in the context of an analysis of variance.

#### *χ^2 ^goodness-of-fit test*

The second tested method was the *χ^2 ^goodness-of-fit *test. This test was performed in Simulation 2 only in order to assess the presence of systematic error in the hit distribution surfaces. It was first recommended in [6] in order to identify systematic error in HTS data. The null hypothesis *H_0_*, here, is that no systematic error is present in the data. If *H_0 _*is true, then the hits are evenly distributed across the well locations and the observed counts of hits *x_ij _*in each row *i *and each column *j *of the hit distribution surface is not significantly different from the expected value calculated as the total counts across the entire surface divided by the number of wells. The rejection region of *H_0 _*is *P(χ^2 ^ > C_α_)>α*, where *C_α _*is the *χ^2 ^*distribution critical value corresponding to the selected *α *parameter (the *α *values equal to 0.01 and 0.1 were tested here) and to the number of degrees of freedom of the model.

For a hit distribution surface with *N_R _*rows and *N_C _*columns, we can assess the presence of systematic error in a given row *r *by computing the test statistic $\chi_{r}^{2}$ by means of Equation 5:

(5) $$\chi_{r}^{2}=\sum_{j=1}^{N_{C}}\frac{{\left(x_{rj}-E\right)}^{2}}{E},$$

where *E *is the total hits count of the whole hit distribution surface divided by the number of wells (*N_R _*× *N_C_*) with the number of degrees of freedom equal to *N_R _*- 1.

Similarly, the columns of the hit distribution surface affected by systematic error can be identified by calculating the test statistic $\chi_{c}^{2}$, using Equation 6 below:

(6) $$\chi_{c}^{2}=\sum_{i=1}^{N_{R}}\frac{{\left(x_{ic}-E\right)}^{2}}{E},$$

where *E *is the total hits count of the whole hit distribution surface divided by the number of wells (*N_R _*× *N_C_*) with the number of degrees of freedom equal to *N_C_*- 1.

The presence of systematic error in the assay can be detected even if systematic error affects particular wells of the assay, not necessarily located in the same row or column. We can achieve it by calculating the test statistic *χ^2 ^*over all well locations of the given hit distribution surface (Equation 7):

(7) $$\chi^{2}=\sum_{i=1}^{N_{R}}\sum_{j=1}^{N_{C}}\frac{{\left(x_{ij}-E\right)}^{2}}{E},$$

where *E *is the total hits count of the whole hit distribution surface divided by the number of wells (*N_R _*× *N_C_*) with the number of degrees of freedom equal to *N_R _*× *N_C _*- 1.

#### Discrete Fourier Transform and Kolmogorov-Smirnov test

The third tested method consisted of the Discrete Fourier Transform (DFT) procedure [9] followed by the Kolmogorov-Smirnov goodness-of-fit test [8]. DFT has been widely used in the frequency analysis of signals and, in particular, for building the signal's density spectrum. The power density spectrum shows the energy contained in each frequency component existing in the signal. In order to apply DFT to HTS data we need first to unroll a plate measurement matrix into a linear sequence of measurements. There are two natural ways to do so: (a) to build the sequence starting by the first row of the plate, followed by the second row, then third one, and so on, and (b) to start by the first column of the plate, followed by the second column, third one, and so on. The analysis of sequences (a) and (b) would allow us to detect column and row effects, respectively. DFT detects frequencies of signals that repeat every two, three, four, and so on, positions in the sequence. DFT calculates the amplitudes of every possible frequency component. Let $y_{k}^{p}$ (1 ≤ *k *≤ *N*) be the power density spectrum generated by the DFT analysis for the plate *p *with *N *wells.

As a second step of this method, we carry out the Kolmogorov-Smirnov test to compute the probability of the density spectrum $y_{k}^{p}$ occurring under the null hypothesis of no effect. The test statistic *D *can be calculated as follows:

(8) $$D=\underset{1\leq k\leq N\times (N_{R}+N_{C})}{\max}\left(F\left(y_{k}^{p}\right)-\frac{k-1}{N},\frac{k}{N}-F\left(y_{k}^{p}\right)\right),$$

where $F\left(y_{k}^{p}\right)$ is defined as the number of values in the density spectrum that are lower than or equal to $y_{k}^{p}$, i.e., $F\left(y_{k}^{p}\right)\equiv \left\|\;\left\{y_{l}^{p},\;1\leq l\leq N,\;y_{l}^{p}<y_{k}^{p}\right\}\;\right\|$. Big values of *D *lead to the rejection of the null hypothesis (i.e., *x_ijp_*'s have been drawn from random normally distributed data). The method consisting of the DFT analysis followed by the Kolmogorov-Smirnov test was included in some commercial software focusing on the detecting systematic error in experimental data (e.g., in *Array Validator *described in [20]).

## Results and discussion

### Simulation 1: Detecting systematic error in individual plates

Simulation 1 consisted of the detection of systematic error on a plate-by-plate basis. Artificial HTS data for three different plate sizes: 96 wells - 8 rows and 12 columns, 384 wells - 16 rows and 24 columns, and 1536 wells - 32 rows and 48 columns were first generated. We started by creating basic error-free datasets for which the well measurements followed a standard normal distribution ~N(0,1). For all datasets the number of plates was set to 1250 - the same as in McMaster *Test assay *[18]. Then, we added 1% of hits to each of the generated basic datasets. The hits were added in such a way that the probability that a given well contained a hit was 1%. All the hit values followed a normal distribution with the parameters ~N(*μ *- 5*SD*, *SD*), where *μ *and *SD *are the mean value and standard deviation of the basic dataset (without hits).

Using these error-free datasets, we generated datasets comprising different types of systematic error, labelled A to E, as reported in Table 1. Systematic error was added only to some of the assay rows (columns, wells). The number of rows (columns, wells) affected by systematic error as well as the indexes of the affected rows, columns and wells were determined randomly for each considered dataset. Six types of error-affected sets were produced for each error-free dataset by varying the standard deviation of systematic error. The following values of the systematic error standard deviation were used: 0, 0.6*SD*, 1.2*SD*, 1.8*SD*, 2.4*SD *and 3.0*SD*, where *SD *is the standard deviation of the basic dataset. The t-test and K-S test were then applied to error-affected data. Both tests produced a binary result for each row and column of each plate: Systematic error was detected or not detected in this row or column. The output was then compared to the information from the data generation phase to determine whether the result of the test was correct.

Cohen's kappa coefficient [22,23] was calculated to estimate the accuracy of both statistical tests. Cohen's kappa is a measure of inter-rater agreement or inter-annotator agreement. The kappa coefficient, which takes into account the agreement occurring by chance, is computed as follows (Equation 9):

(9) $$\kappa =\frac{\mathrm{Pr}(a)-\mathrm{Pr}(e)}{1-\mathrm{Pr}(e)},$$

where Pr(*a*) is the relative observed agreement among raters (i.e., statistical tests in our study) and Pr(*e*) is the hypothetical probability of chance agreement. If the raters are in complete agreement, then *κ *= 1. If there is no agreement among the raters, other than what would be expected by chance, then *κ *≤ 0.

In our HTS context, Pr(*a*) and Pr(*e*) were calculated as follows: $\mathrm{Pr}(a)=\frac{TP+TN}{P\times (N_{R}+N_{C})}$ and $\mathrm{Pr}(e)=\frac{\left(TP+FN)\times (TP+FP)+(TN+FN)\times (TN+FP\right)}{{\left(P\times (N_{R}+N_{C})\right)}^{2}}$, where *P *is the number of plates in the assay, *N_R _*and *N_C_*, are, respectively, the number of rows and columns per plate, *TP *(*true positives*) is the sum of the numbers of rows and columns where systematic error was added during the data generation and then detected by the test, *FP *(*false positives*) is the sum of the numbers of rows and columns where systematic error was not added but detected by the test, *TN *(*true negatives*) is the sum of the numbers of rows and columns where systematic error was not added and not detected by the test, and *FN *(*false negatives*) is the sum of the numbers of rows and columns where systematic error was added but not detected by the test.

For all generated variants of error-affected data, 500 different sets were created. The averages of obtained Cohen's kappa coefficients are represented in Figures 2, 3 and 4 (for the 96, 384 and 1536-well plates, respectively). Also, the sensitivity (Figures 1SM, 2SM and 3SM, see the section Supplementary Materials available in Additional file 1), specificity (Figures 4SM, 5SM and 6SM) and success rate (Figures 13SM, 14SM and 15SM) of the two tests are depicted. The sensitivity and specificity of the two tests were calculated as follows (Equations 10):

(10) $$Sensitivity=\frac{TP}{TP+FN},\;Specificity=\frac{TN}{TN+FP}.$$

Since datasets of types C and E did not contain row or column systematic error, the sensitivity and Cohen's kappa coefficient of both competing statistical tests for these data were undefined (i.e., *TP *= *FN *= 0 for these data types).

The kappa coefficient curves in Figures 2, 3 and 4 show that the t-test clearly outperforms DFT followed by the K-S test for all selected sizes of systematic error, confidence levels and plate sizes. The accuracy of the t-test grows as the size of systematic error increases. It also grows slightly as the plate size increases. The accuracy of the K-S test remains very low and usually varies between 0.0 and 0.1, thus suggesting a very poor systematic error recovery by this test. Figures 13SM, 14SM and 15SM indicate that the success rate of the t-test is largely independent of the systematic error variance and remains very steady for all tested types of systematic error and plate sizes. In contrast, the success rate of the K-S test decreases as the standard deviation of systematic error increases. The performance of the K-S test is also affected by the size of the plate (Figures 2, 3 and 4). The K-S test success rate decreases significantly, and often falls below 50%, for larger plates (Figure 15SM). The chosen confidence level *α *affects the accuracy of both statistical tests. For instance, the use of *α *= 0.1 generally causes a decrease in the kappa coefficient (the decrease of 0.2 on average, see Figures 2, 3 and 4) and in the success rate (the decrease of 10% on average, see Figures 13SM, 14SM and 15SM) of the t-test, when compared to *α *= 0.01. The sensitivity charts (Figures 1SM, 2SM and 3SM) show that the increase in the variance of systematic error leads to the increase in sensitivity of both tests. In terms of sensitivity, the t-test outperforms the K-S test for all data types and all sizes of systematic error, the only exception being large plates tested with the confidence level *α *= 0.1 (Figure 3SM).

Similarly to real HTS assays, our artificially generated datasets had systematic error in only a few rows and/or columns. They contained many negative and only a few positive samples. Such an imbalance between positive and negative samples implies that the overall accuracy of the tests will depend much more on the test specificity than on its sensitivity. Figures 4SM, 5SM and 6SM confirm this observation - most of the specificity charts resemble the corresponding success rate charts (see Figures 13SM, 14SM and 15SM).

### Simulation 2: Detecting systematic error on hit distribution surfaces

The second simulation, Simulation 2, consisted of the detection of systematic error on the hit distribution surfaces. The recommendation to use statistical tests to examine hit distribution surfaces of experimental HTS assays was first formulated in [6], in the case of the *χ^2 ^*test. In Simulation 2, we also considered artificially generated assays with plates of three different sizes (i.e., 96-, 384- and 1536-well plates as well as 1250-plate assays) with the measurements following the standard normal distribution. From every basic dataset we generated 6 error-free datasets comprising 0.5%, 1%, 2%, 3%, 4% and 5% of hits. All the hit values followed a normal distribution with the parameters ~N(*μ *- 5*SD*, *SD*). Using the error-free datasets, we generated assays containing different types of systematic error (i.e., from A to E). Systematic error, added to some of the assay rows (columns, wells) only, followed the normal distribution with the mean value of 0 and the standard deviation of 1.2*SD*. For each such an assay, we calculated its hit distribution surface for the hit selection threshold of *μ*-3*σ*. Then we applied, in turn, the t-test, and the K-S and *χ^2 ^*goodness-of-fit tests to detect the presence of systematic error.

**Figure 5:**
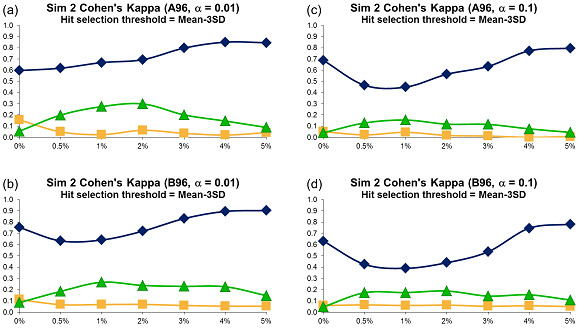
**Simulation 2, Plate Size: 96 wells, Cohen's Kappa vs Hit Percentage**. Systematic error size: 10% (at most 2 columns and 2 rows affected). First column: cases (a) - (b): *α *= 0.01; Second column: cases (c) - (d): *α *= 0.1. Systematic Error Detection Tests: (◆) t-test, (■) K-S test and (▲)*χ^2 ^*goodness-of-fit test.

**Figure 6:**
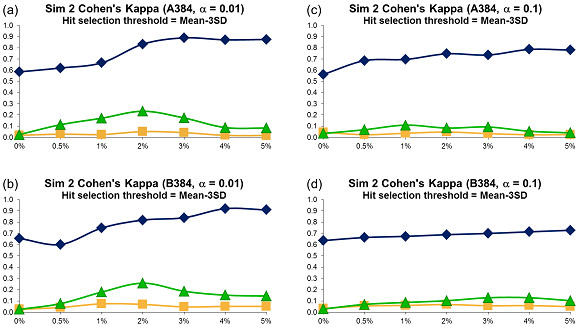
**Simulation 2, Plate Size: 384 wells, Cohen's Kappa vs Hit Percentage**. Systematic error size: 10% (at most 4 columns and 4 rows affected). First column: cases (a) - (b): *α *= 0.01; Second column: cases (c) - (d): *α *= 0.1. Systematic Error Detection Tests: (◆) t-test, (■) K-S test and (▲)*χ^2 ^*goodness-of-fit test.

**Figure 7:**
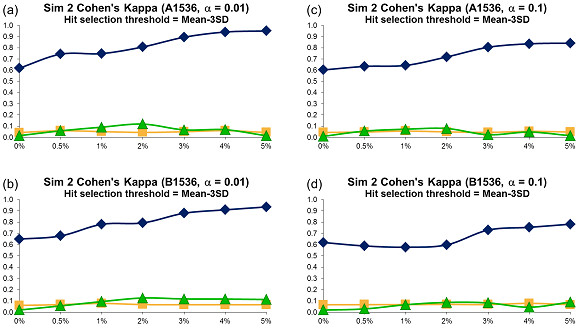
**Simulation 2, Plate Size: 1536 wells, Cohen's Kappa vs Hit Percentage**. Systematic error size: 10% (at most 8 columns and 8 rows affected). First column: cases (a) - (b): *α *= 0.01; Second column: cases (c) - (d): *α *= 0.1. Systematic Error Detection Tests: (◆) t-test, (■) K-S test and (▲)*χ^2 ^*goodness-of-fit test.

For each error variant, 500 different datasets were generated and the averages of obtained Cohen's kappa coefficients were plotted in Figures 5, 6 and 7. The sensitivity and specificity of the three tests were depicted in Figures 7SM to 12SM, and the success rate in Figures 16SM, 17SM and 18SM. The hit distribution surfaces for the assays of types C, D and E (these assays don't contain systematic error that repeats along all assay plates) cannot be used to retrace row or column systematic error. Hence, the sensitivity and Cohen's kappa coefficient for datasets of types C, D and E were undefined.

The kappa coefficient curves presented in Figures 5, 6 and 7 illustrate that the t-test clearly outperforms the *χ^2 ^*goodness-of-fit test as well as the combination of DFT and the K-S test for all selected sizes of systematic error, confidence levels and plate sizes. The accuracy of the t-test generally grows as the size of systematic error increases, but this trend is not as steady as in Simulation 1: The curve's minimum is not always associated with the lowest systematic noise (e.g., see cases *c *and *d *in Figure 5). The kappa values for the *χ^2 ^*and K-S tests usually varies between 0.0 and 0.25, thus suggesting a poor systematic error recovery provided by both of them. As in Simulation 1, the success rate of the t-test is largely independent of the systematic error variance (Figures 16SM, 17SM and 18SM). Moreover, the success rate of the t-test varies between 90 and 100% in the most of simulated experiments. At the same time, the accuracy of the K-S test is extremely low in almost all of the considered situations. The success rate analysis of the *χ^2 ^*goodness-of-fit test suggests that this test follows different patterns for different types of data. For datasets of types D and E, whose hit distribution surfaces did not contain systematic error, the accuracy of the *χ^2 ^*test is very close to that of the t-test (Figures 16SM, 17SM and 18SM, cases *d*, *e, i *and *j*). However, for the datasets that contained row and/or column systematic error and well systematic error, the success rate of the *χ^2 ^*goodness-of-fit test is significantly lower than that of the t-test (Figures 16SM, 17SM and 18SM, cases *a *to *c *and *f *to *h*) and shows a tendency to deteriorate when the percentage of hits in the data increases. The sensitivity patterns shown in Figures 7SM, 8SM and 9SM demonstrate that the sensitivity of the three statistical tests grows as the percentage of hits contained in the data increases. Similarly to Simulation 1, choosing a bigger value of *α *led to a decrease in the accuracy of all tests.

### Application to the McMaster data

As a final step in our study we applied the three discussed systematic error detection tests on real HTS data. We examined the impact that the presented methodology would have on the hit selection process in McMaster Data Mining and Docking Competition *Test assay *[18]. Similarly to Simulations 1 and 2 carried out with artificial data, we performed two types of analysis. First, we studied the raw HTS measurements, and then calculated and analyzed the hit distribution surfaces of *Test assay*.

We carried out the t-test on every plate of *Test assay*, scanning all rows and columns of each plate for the presence of systematic error. We performed the calculation for several confidence levels including: *α *= 0.01, 0.05, 0.1 and 0.2. In each case, we counted the number of rows and columns in which the test reported the presence of systematic error and also the number of plates in which at least one row or column contained systematic error. The collected results are presented in Table 2.

**Table 2:** Number of rows, columns and plates (where at least one row or column contains systematic error) of McMaster *Test assay *in which the t-test reported the presence of systematic error, depending on the *α *parameter

***α***	Plates	Rows	Rows %	Columns	Columns %
0.01	159	76	0.76%	94	0.75%

0.05	814	575	5.76%	606	4.86%

0.1	1121	1148	11.50%	1296	10.38%

0.2	1241	2242	22.46%	2583	20.70%

Only 8 rows and 10 columns of McMaster *Test assay *were examined because the first and twelfth columns of the (8 by 12) plates were used for controls.

The obtained results suggest that the number of positives for the row and column effects is almost exactly what we would expect by chance (e.g., approximately 1% when we used *α *= 0.01, 5 % when we used *α *= 0.05, etc.). This means that there is no statistical evidence of bias for columns and rows in McMaster *Test assay*.

For comparative purposes, we corrected the raw McMaster data using the B-score method in all plates where systematic error was detected by the t-test. Unlike the artificially generated data used in the simulation study, McMaster *Test assay *contained replicated plates - every compound of the assay was screened twice [18]. We adjusted our hit selection procedure to search for *average hits*. Thus, we first calculated the average of the two compound measurements and then used it in the hit selection process. If systematic error was detected only in first plate and, therefore, corrected using the B-score method, then the residuals produced by B-score were incomparable with the values of the second (i.e., replicated) plate. In order to make the measurements in both plates comparable, we normalized both plates by means of the Z-score method prior to calculating the average compound activity. Using the corrected dataset, we determined the assay hits for two hit selection thresholds: *μ-*3*SD*- the most popular hit cutting threshold employed in HTS, and *μ-*2.29*SD*- the threshold used by the McMaster competition organizers to identify the original 96 average hits. The obtained results are reported in Tables 3 and 4, respectively. A comparison between the original set of hits and the newly selected hits is also made in these tables. In fact, these tables report how many of the original hits remained hits, how many of them were removed and how many new hits were selected. For the threshold *μ-*3*SD*, only about half of the original hits were preserved, whereas for the threshold *μ-*2.29*SD *about four times more hits were selected for the B-score corrected data. The presented results demonstrate how significantly the selected error correction method and confidence level *α *can affect the hit selection process in experimental HTS.

**Table 3:** Number of hits selected in McMaster *Test assay *for the *μ-*3*SD *threshold after the application of the B-score correction, depending on the *α *parameter

***α***	Original hits	Obtained hits	Preserved hits	Added hits	Removed hits
0.01	96	123	57	66	39

0.05	96	125	55	70	41

0.1	96	126	52	74	44

0.2	96	130	55	75	41

The t-test was carried out to detect systematic error.

**Table 4:** Number of hits selected in McMaster *Test assay *for the *μ-*2.29*SD *threshold (i.e., threshold used by the McMaster competition organizers to select the 96 original average hits) after the application of the B-score correction, depending on the *α *parameter

***α***	Original hits	Obtained hits	Preserved hits	Added hits	Removed hits
0.01	96	357	79	278	17

0.05	96	419	79	340	17

0.1	96	411	79	332	17

0.2	96	417	76	341	20

The t-test was carried out to detect systematic error.

In our second experiment, we computed and analyzed the hit distribution surfaces of McMaster *Test assay *for the hit selection thresholds: *μ-*3*SD *and *μ-*2*SD*. We assessed the presence of systematic error in the assay by applying the three discussed systematic error detection tests: t-test, K-S test and *χ^2 ^*goodness-of-fit test. All three tests detected the presence of systematic error in both surfaces for both considered confidence levels *α *= 0.01 and 0.1. While the hit distribution surface is useful for detecting the presence of overall bias, it does not capture the variability of the bias on a plate-by-plate basis.

**Figure 8:**
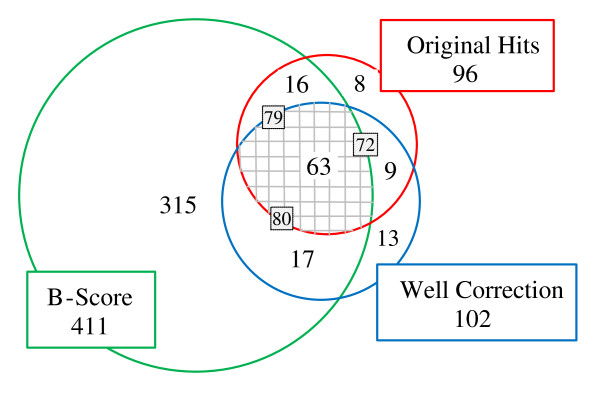
**Intersections between the original set of hits (96 hits in total) and the sets of hits obtained after the application of the *B-score *(411 hits in total; the method was carried out only on the plates where systematic error was detected) and *Well correction *methods (102 hits in total) computed for McMaster *Test assay***. The *μ- 2.29SD *hit selection threshold was used to select hits.

Finally, we also applied the Well correction method to remove systematic error from McMaster *Test assay*. After Well correction was performed, the hit selection was carried out again for the hit selection thresholds: *μ-*3*SD *and *μ-*2.29*SD*. Table 5 reports the comparative results of the two hit selections. When analyzing the obtained hits for the *μ-*2.29*SD *threshold, one can notice that 24 of the original hits were not detected and, at the same time, 30 new compounds were selected as hits. Figure 8 presents a summary of our experiments conducted with McMaster *Test assay*. The pairwise intersections between the three obtained sets of hits are presented. The dashed grey area in the middle represents the intersections between the three hit sets and thus defines the *consensus hits *for McMaster *Test assay*. The results provided by the B-score method (414 hits in total) shows that this data correction procedure tends to overestimate, at least when compared to Z-score and Well correction, the number of hit compounds. On the other hand, the results provided by the Well correction method suggest that about one third of the original hits could be, in fact, false positives and that about the same percentage of false negatives could be ignored if systematic error present in the raw McMaster data is not identified and removed adequately.

**Table 5:** Number of hits selected in McMaster *Test assay *for the *μ-*3*SD *and *μ-*2.29*SD *thresholds after the application of the Well Correction method

Threshold	Original hits	Obtained hits	Preserved hits	Added hits	Removed hits
*μ-*3*SD*	96	26	26	0	70

*μ-*2.29*SD*	96	102	72	30	24

## Conclusions

In this article we discussed and tested three methods for detecting the presence of systematic error in experimental HTS assays. We conducted a comprehensive simulation study with artificially generated HTS data, constructed to model a variety of real-life situations. The variants of each dataset, comprising different hit percentages and various types and levels of systematic error, were examined. The experimental results show that the method performances depend on the assay parameters - plate size, hit percentage, and type and variance of systematic error. We found that the simplest, and computationally fastest method, the t-test, outperformed the Kolmogorov-Smirnov (K-S) and *χ^2 ^*goodness-of-fit tests in most of the practical situations. The t-test demonstrated a high robustness when applied on a variety of artificial datasets. The success rate of the t-test was, in most situations, well above 90%, regardless the plate size, noise level and type of systematic error, while the values of Cohen's kappa coefficient computed for this test suggested its superior performance especially in the case of large plates and high level of systematic noise. We can thus recommend the t-test as a method of choice in experimental HTS. On the contrary, advocated in some works [20,21] Discrete Fourier Transform followed by the K-S test yielded very disappointing results. Moreover, the latter technique required a lot of computational power but provided the worst overall performance among the three competing statistical procedures. The K-S test can still be used to examine HTS data located in small plates (i.e., 96-well plates), but we strongly recommend not using it for the analysis or large plates (i.e., 384 and 1536-well plates) and hit distribution surfaces. The main reason for such a disappointing performance of the K-S test is it that was applied, as recommended in [20], on the data already transformed by the Discrete Fourier method. Figure 19SM presents an example of data from one of the simulated 96-well plates before and after the application of Discrete Fourier Transform. The raw data followed a normal distribution and contained random error only (i.e., systematic error was not added). The raw data did not deviate from the normal distribution, as shown both graphically (Figure 19SMa) and by the K-S test (*KS *= 0.03, *p *= 0.5). However, after the application of Discrete Fourier Transform, the data deviate from normality as shown in the graph (Figure 19SMa) and by the K-S test (*KS *= 0.06, *p *= 0.0018). The third method, the *χ^2 ^*goodness-of-fit test suggested in [6], can be employed to assess hit distribution surfaces for the presence of systematic error. In general, its performances were lower than those of the t-test and were very sensitive to the type of systematic error as well as to its variance. The *χ^2 ^*goodness-of-fit test could be recommended, especially to analyze HTS assays with small plate sizes, but we suggest carrying out the t-test as well to confirm its results.

In addition to the experiments with simulated data, we applied the three discussed systematic error detection tests to real HTS data. Our goal was to evaluate the impact of systematic error on the hit selection process in experimental HTS. The obtained results (see Tables 2-5 and Figure 8) confirm the following fact: If raw HTS data are not treated properly for eliminating the effect of systematic error, then many (e.g., about 30% of hits in the case of McMaster *Test assay*, as reported in Table 5) of the selected hits may be due to the presence of systematic error and, at the same time, many promising compounds may be missed during hit selection. A special attention should be paid to control the results of aggressive data normalization procedures, such as B-score, that could easily do more damage by introducing biases in raw HTS data and, therefore, lead to the selection of many false positive hits even in the situations when the data don't contain any kind of systematic error.

Our general conclusion is that a successful assessment of the presence of systematic error in experimental HTS assays is achievable when the appropriate statistical methodology is used. Namely, the t-test should be carried out by HTS researchers to pre-process raw HTS data. This test should help improve the "quality" of selected hits by discarding many potential false positives and suggesting new, and eventually real, active compounds. The t-test should be used in conjunction with data correction techniques such as: Well correction [5,6], when row or column systematic error (detected by the test) repeats across all plates of the assay, and B-score [3] or trimmed-mean polish score [7], when systematic error varies across plates. Thus, we recommend adding an extra preliminary systematic error detection and correction step in all HTS processing software and using consensus hits in order to improve the overall accuracy of HTS analysis.

## Authors' contributions

PD and VM designed the study. PD implemented the statistical tests and carried out the simulations. VM and RN supervised the project and coordinated the methodological development.

## Supplementary Material

Additional file 1 **Supplementary Materials**. Additional file 1 includes Supplementary Materials for the article. This file contains Figures 1SM to 19SM presenting additional simulation results (Figures 1SM to 18SM) and an example of data distribution before and after the application of the Discrete Fourier Transform (DFT) method (Figure 19SM). Click here for file
