# Supplementary material for: Systematic error detection in experimental high-throughput screening
Source: BMC Bioinformatics. 2011 Jan 19;12:25. doi: 10.1186/1471-2105-12-25 (PMC3034671; doi:10.1186/1471-2105-12-25)
Supplement: Additional file 1 — Supplementary Materials. Additional file 1 includes Supplementary Materials for the article. This file contains Figures 1SM to 19SM presenting additional simulation results (Figures 1SM to 18SM) and an example of data distribution before and after the application of the Discrete Fourier Transform (DFT) method (Figure 19SM). [file 1471-2105-12-25-S1.DOC]

# Supplementary materials

## Figure 1SM - Simulation 1, Plate Size: 96 wells – Sensitivity (True Positive Rate)

Systematic error size: 10% (at most 2 columns and 2 rows affected). First column: cases (a) - (c): *α* = 0.01; Second column: cases (d) - (f): *α* = 0.1. Systematic Error Detection Tests: (⧫) t-test and (◼) K-S test.


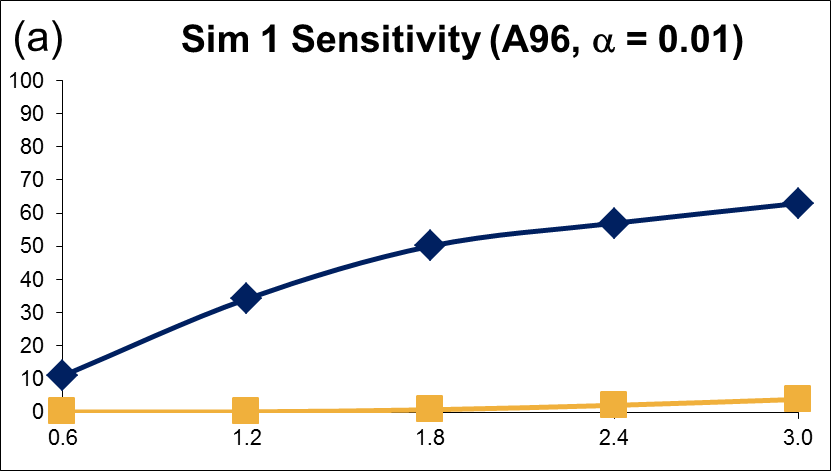

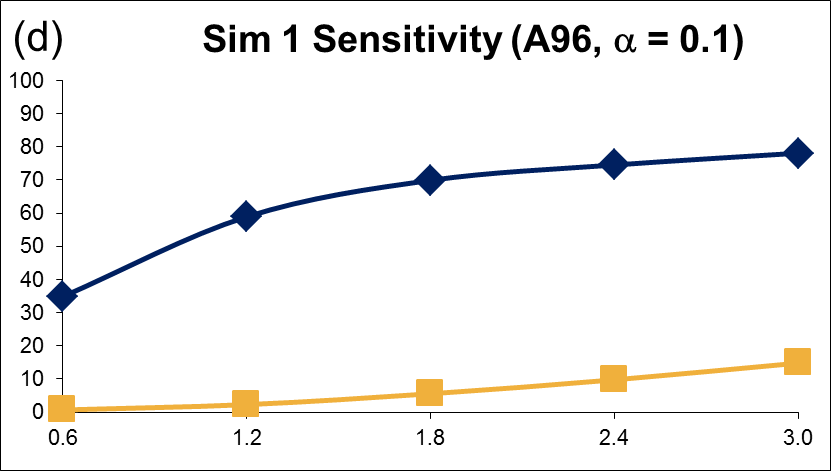


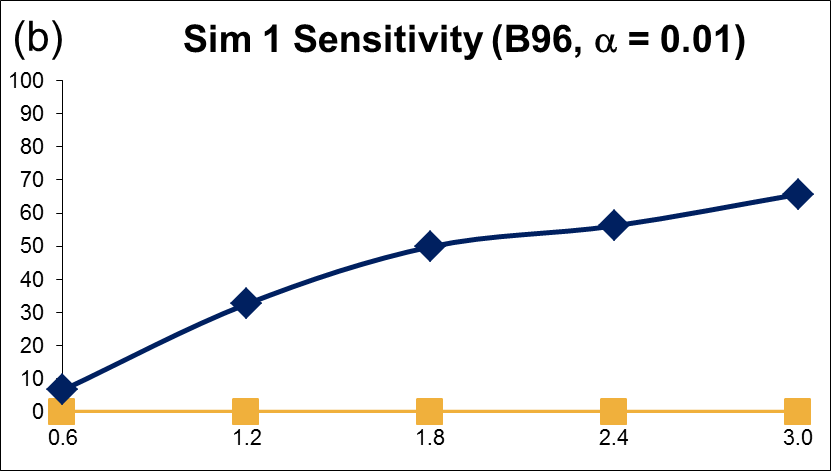

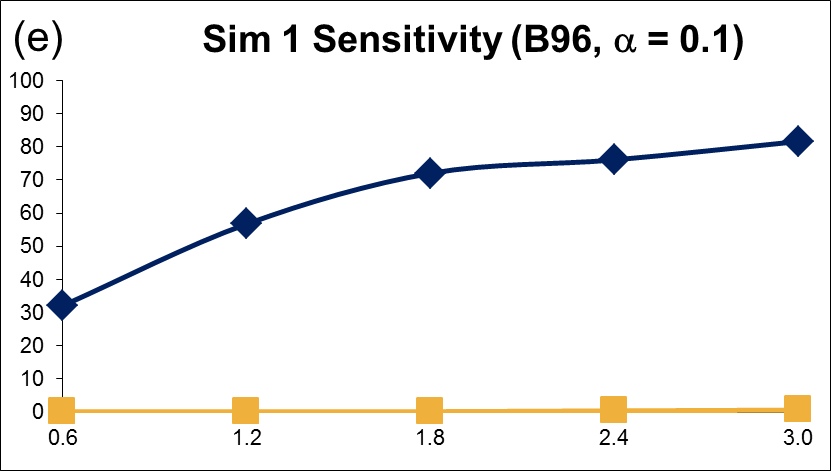


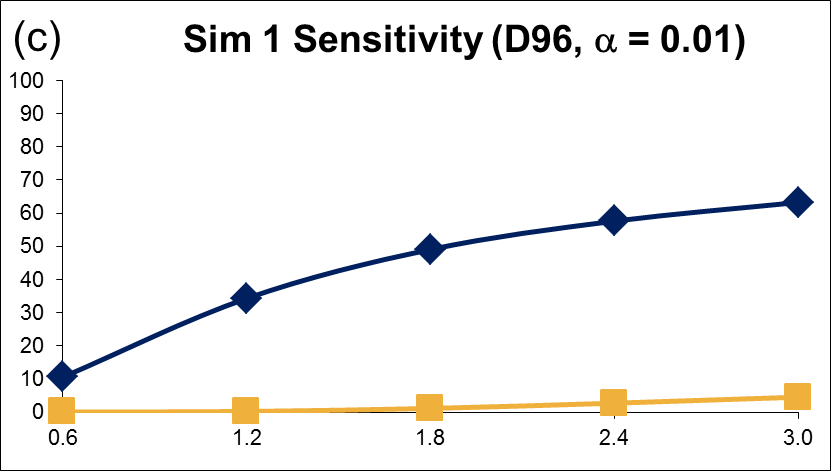

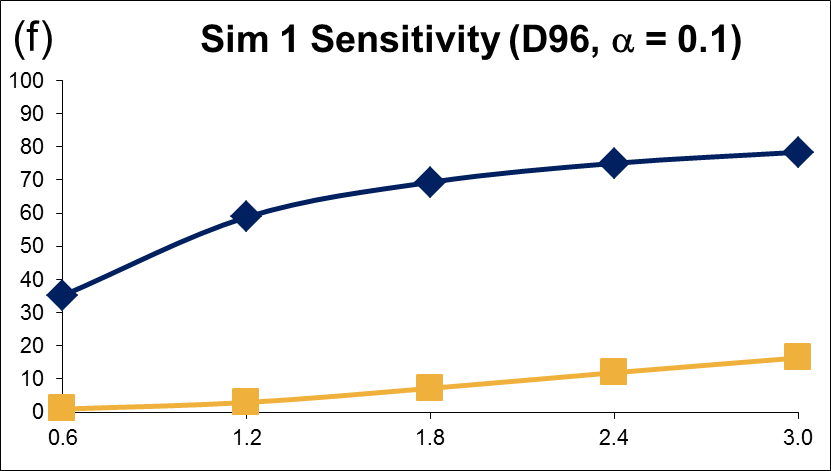


## Figure 2SM - Simulation 1, Plate Size: 384 wells – Sensitivity (True Positive Rate)

Systematic error size: 10% (at most 4 columns and 4 rows affected). First column: cases (a) - (c): *α* = 0.01; Second column: cases (d) - (f): *α* = 0.1. Systematic Error Detection Tests: (⧫) t-test and (◼) K-S test.


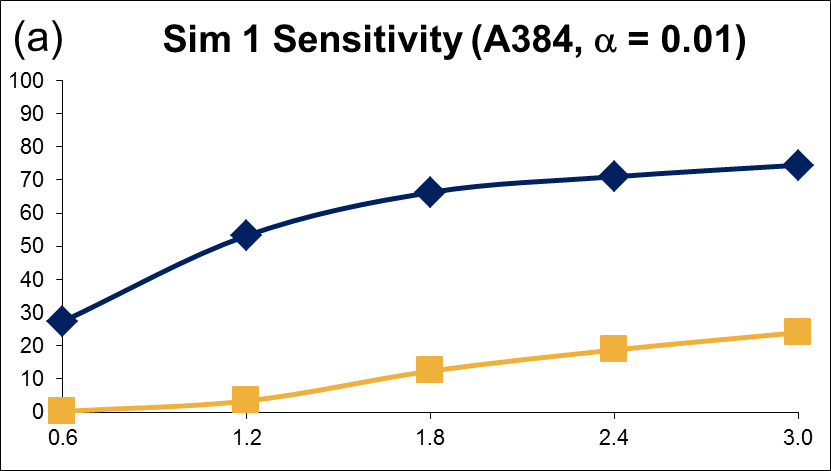

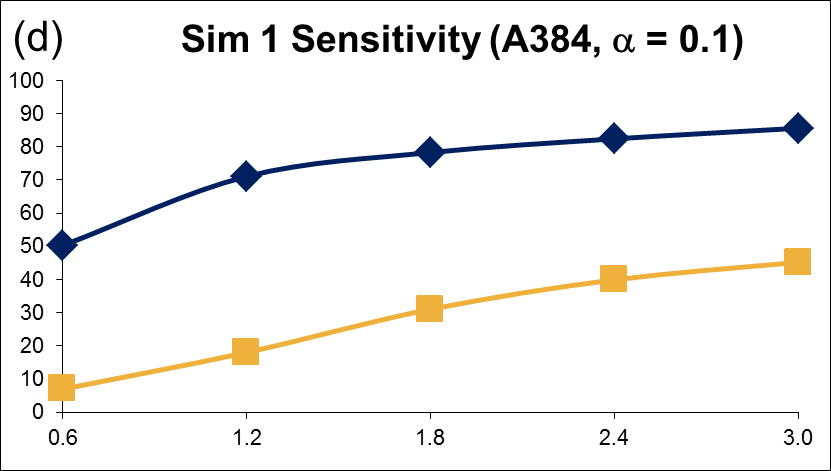


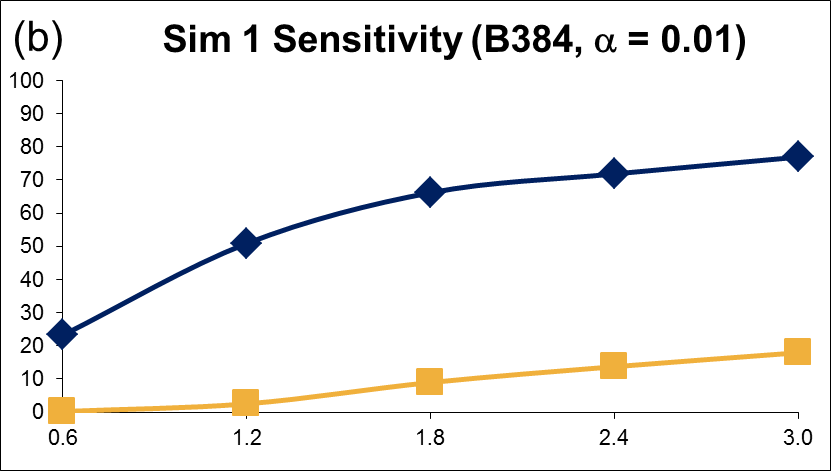

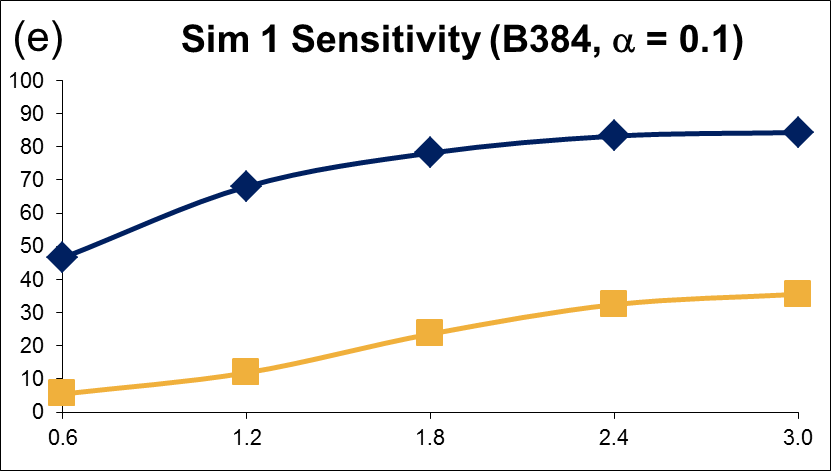


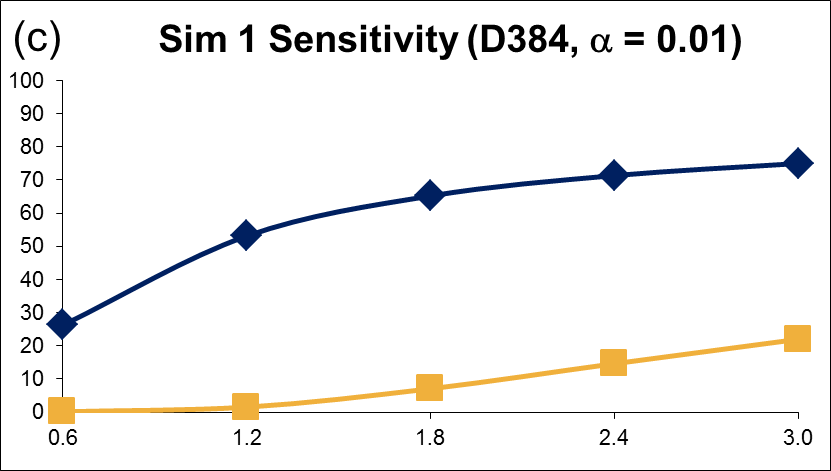

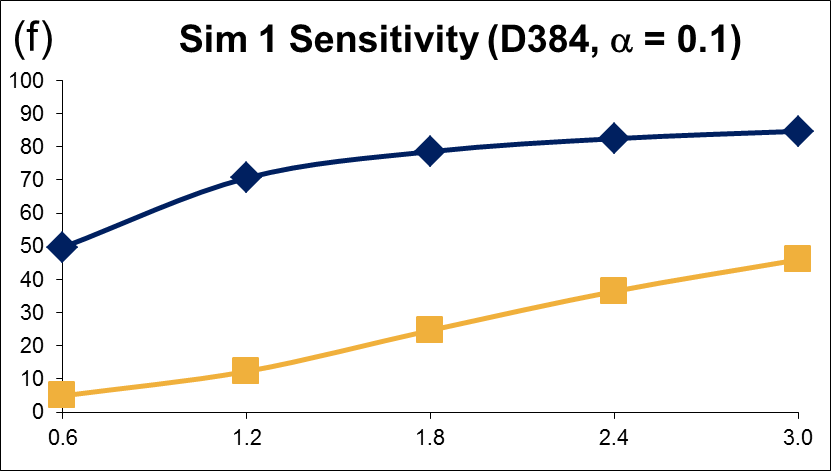


## Figure 3SM - Simulation 1, Plate Size: 1536 wells – Sensitivity (True Positive Rate)

Systematic error size: 10% (at most 8 columns and 8 rows affected). First column: cases (a) - (c): *α* = 0.01; Second column: cases (d) - (f): *α* = 0.1. Systematic Error Detection Tests: (⧫) t-test and (◼) K-S test.


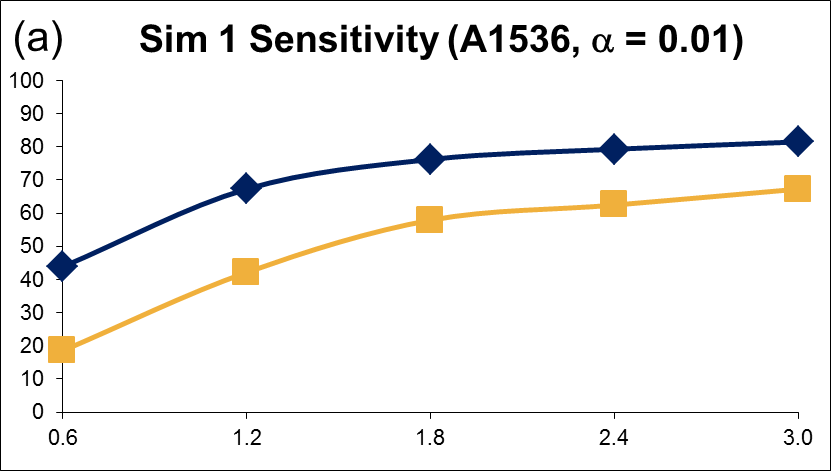

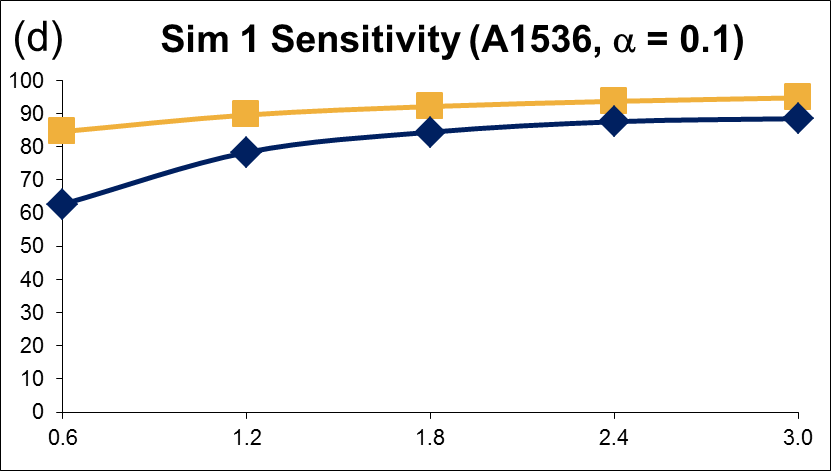


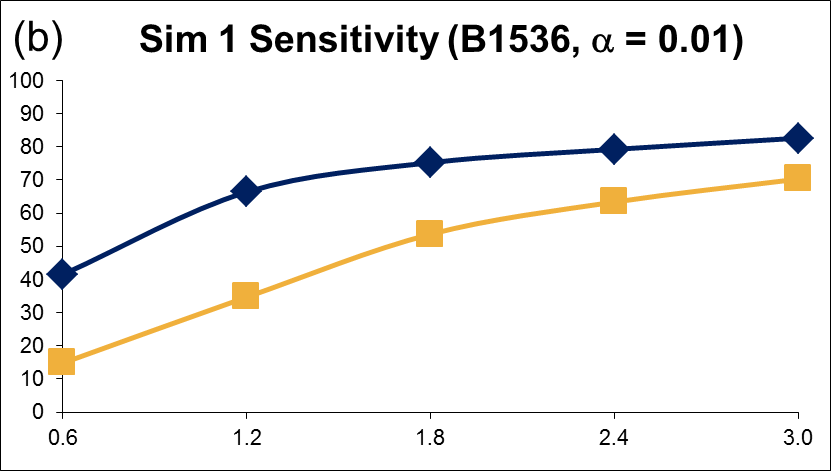

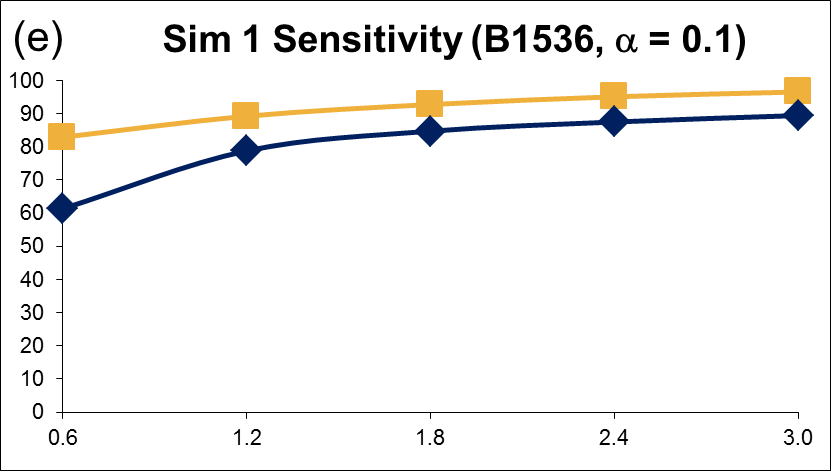


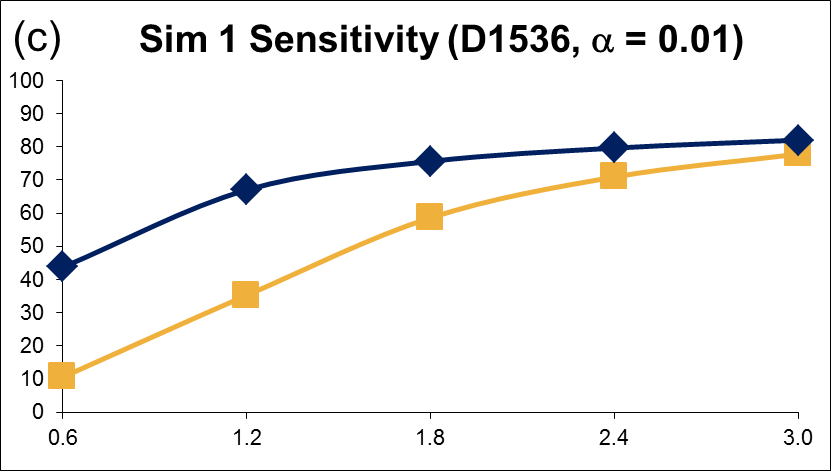

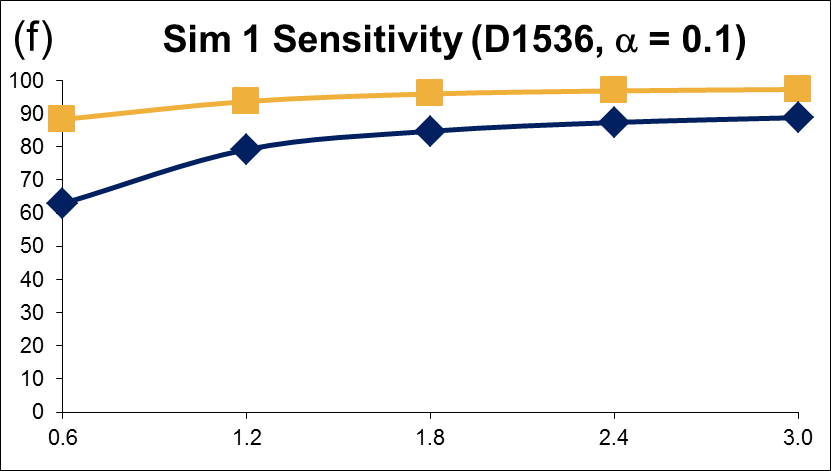


## Figure 4SM - Simulation 1, Plate Size: 96 wells – Specificity (True Negative Rate)

Systematic error size: 10% (at most 2 columns and 2 rows affected). First column: cases (a) - (e): *α* = 0.01; Second column: cases (f) - (j): *α* = 0.1. Systematic Error Detection Tests: (⧫) t-test and (◼) K-S test.


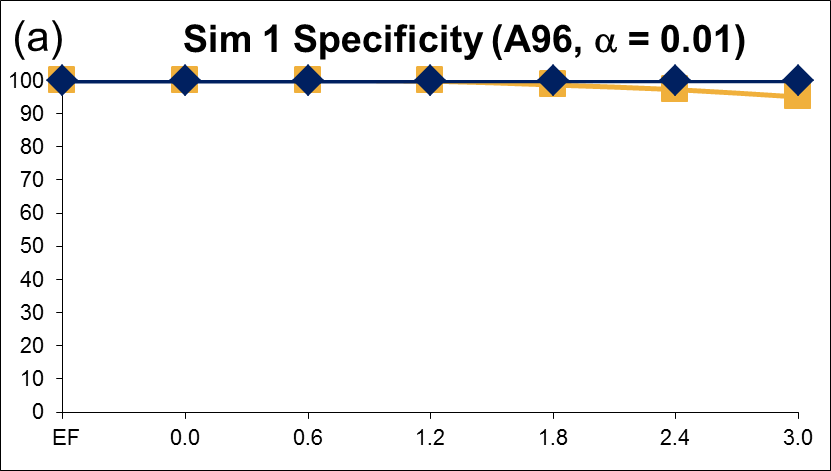

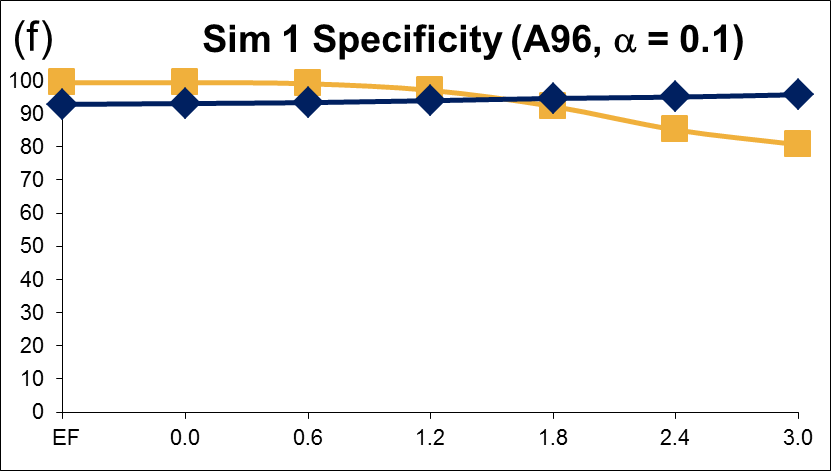


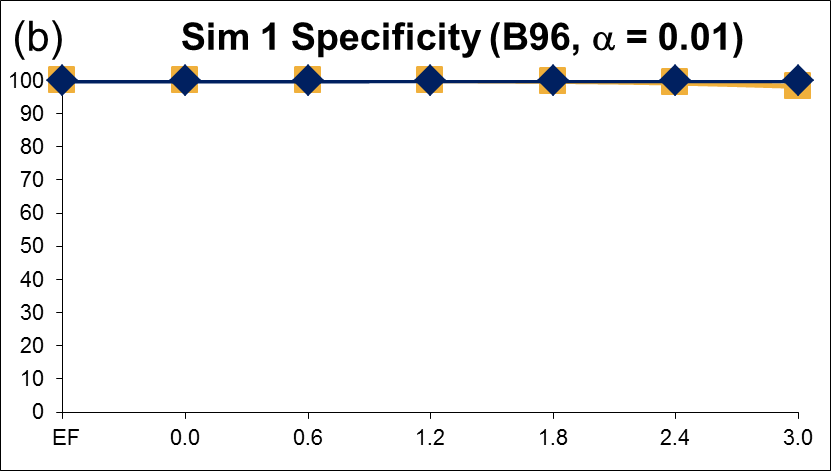

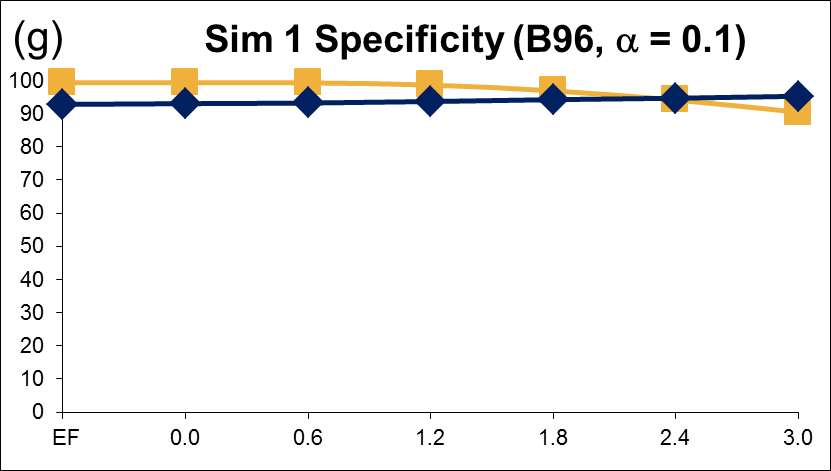


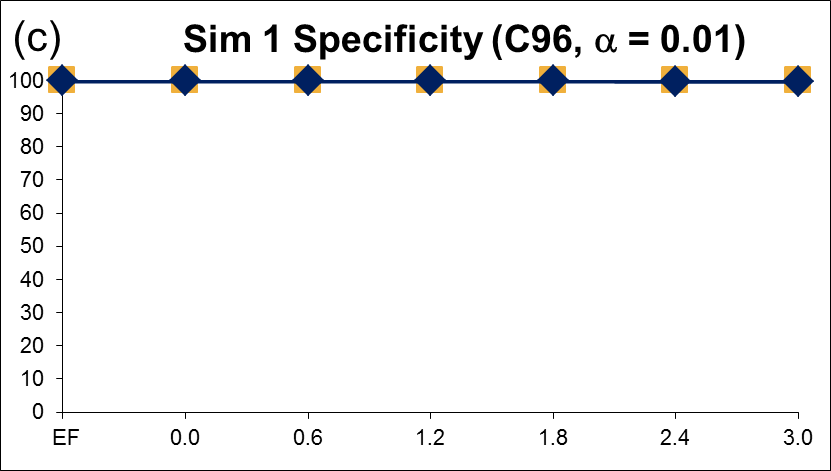

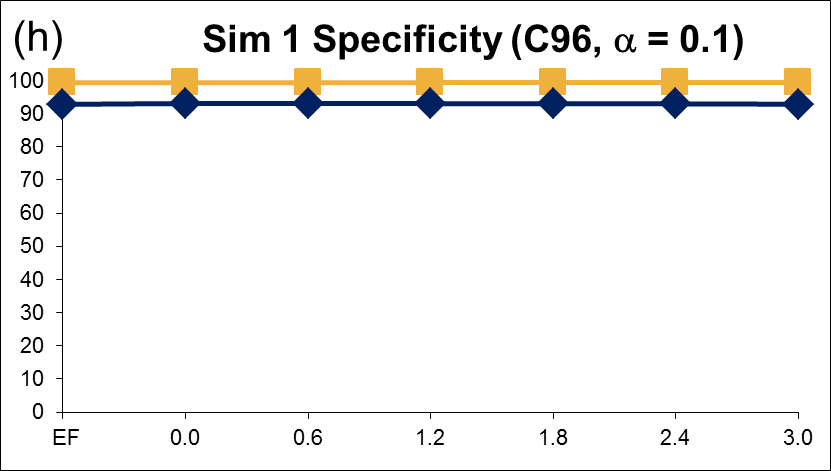


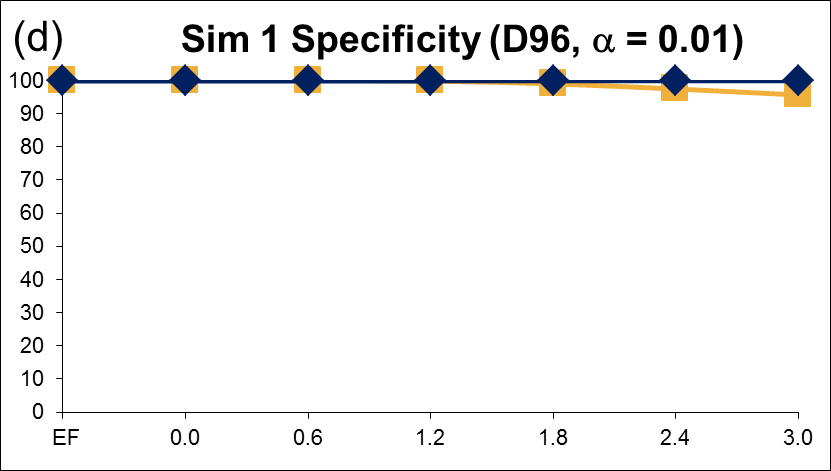

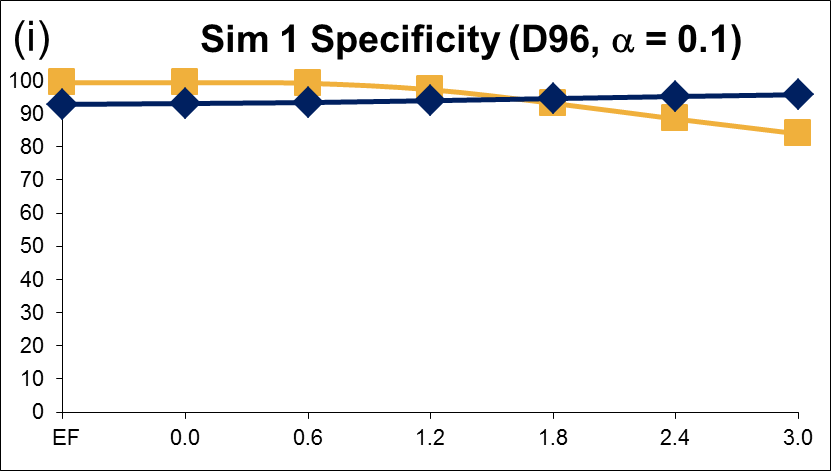


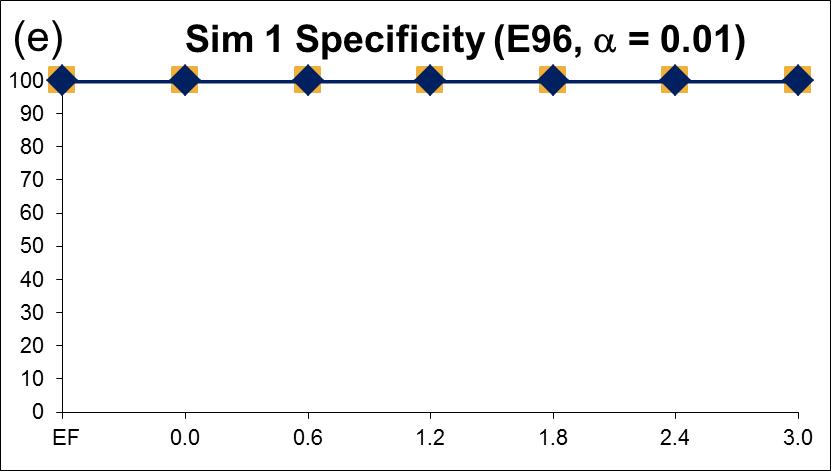

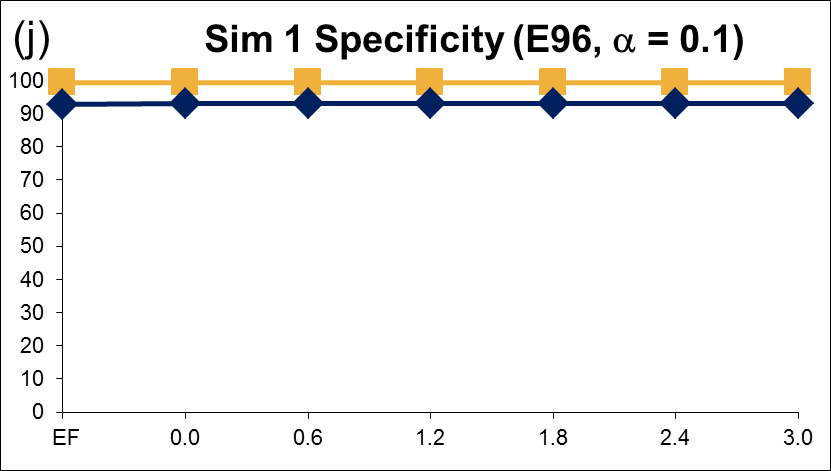


## Figure 5SM - Simulation 1, Plate Size: 384 wells – Specificity (True Negative Rate)

Systematic error size: 10% (at most 4 columns and 4 rows affected). First column: cases (a) - (e): *α* = 0.01; Second column: cases (f) - (j): *α* = 0.1. Systematic Error Detection Tests: (⧫) t-test and (◼) K-S test.


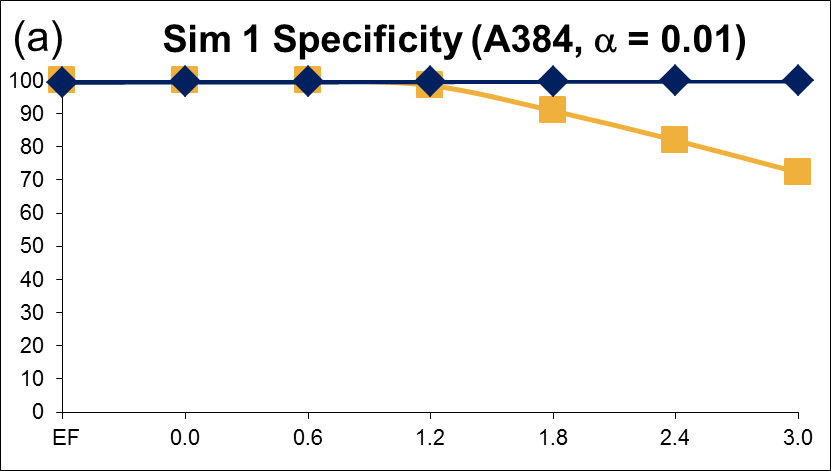

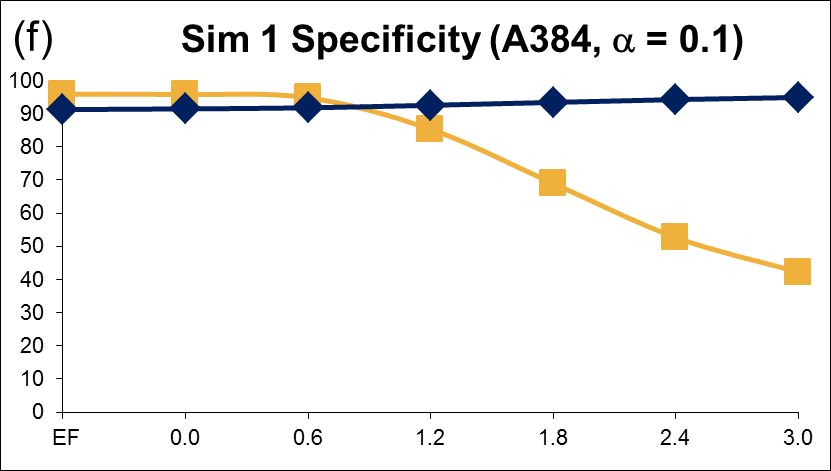


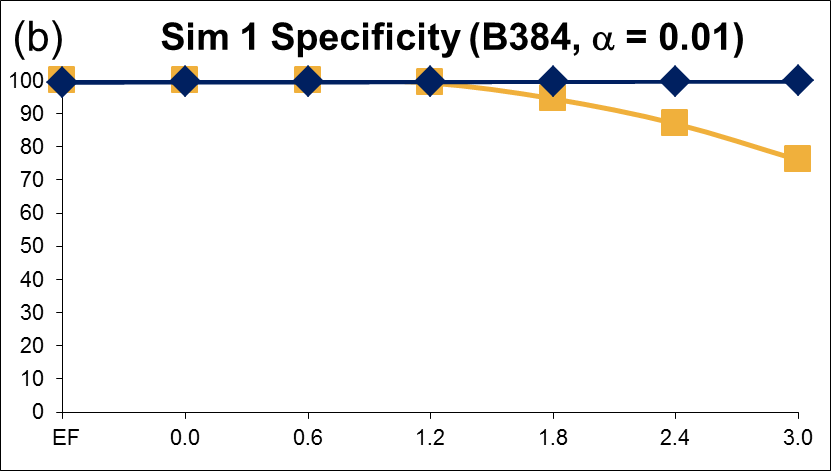

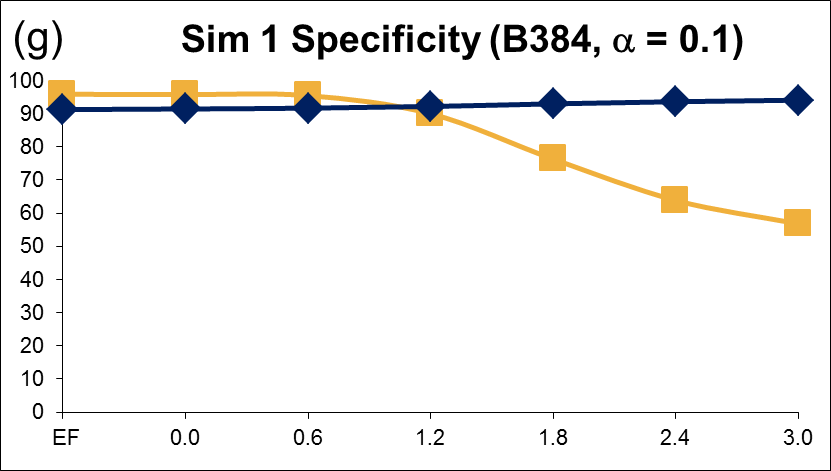


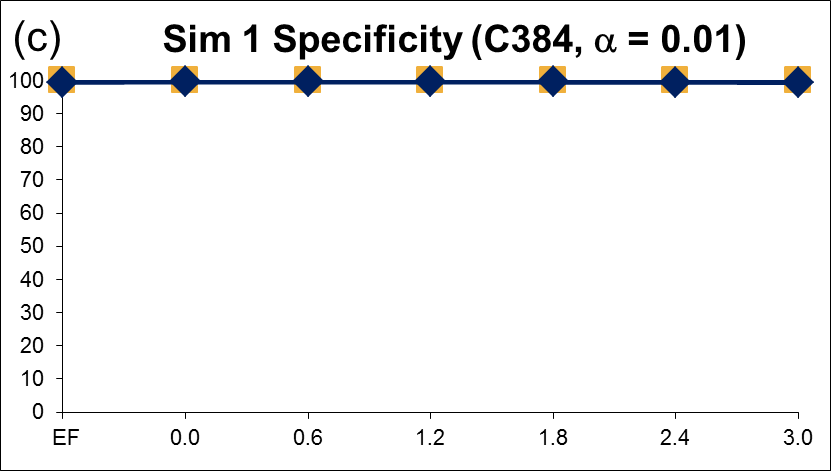

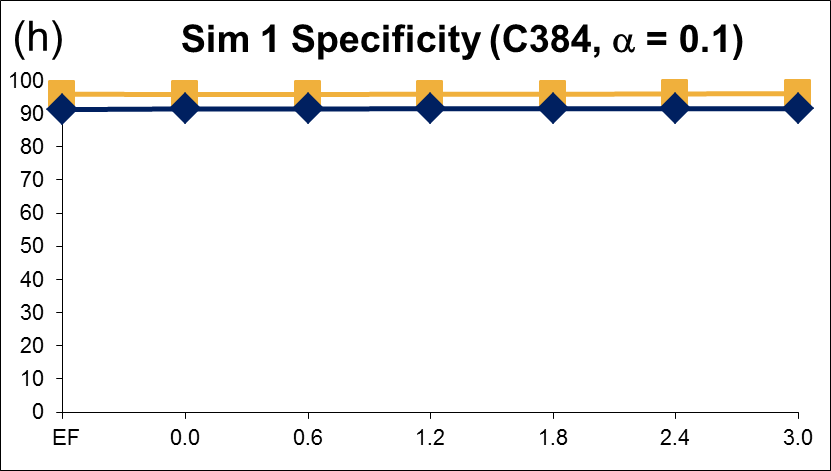


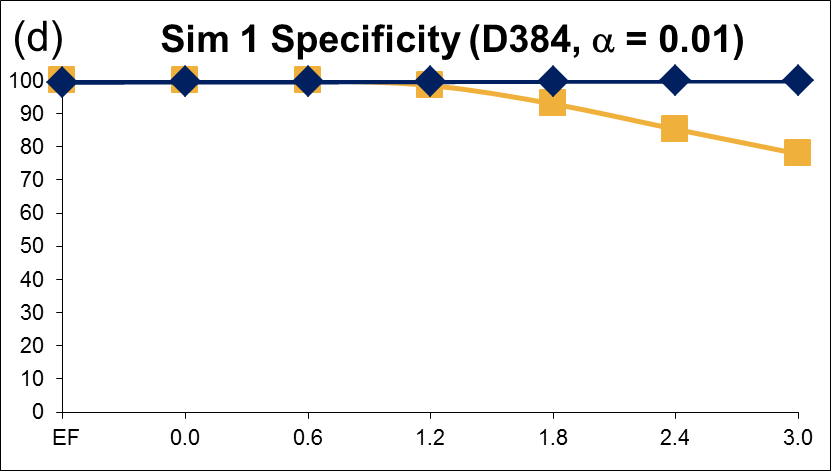

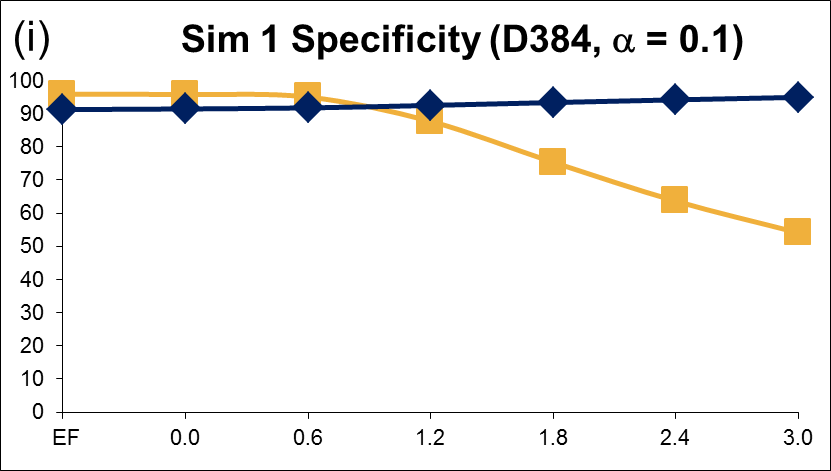


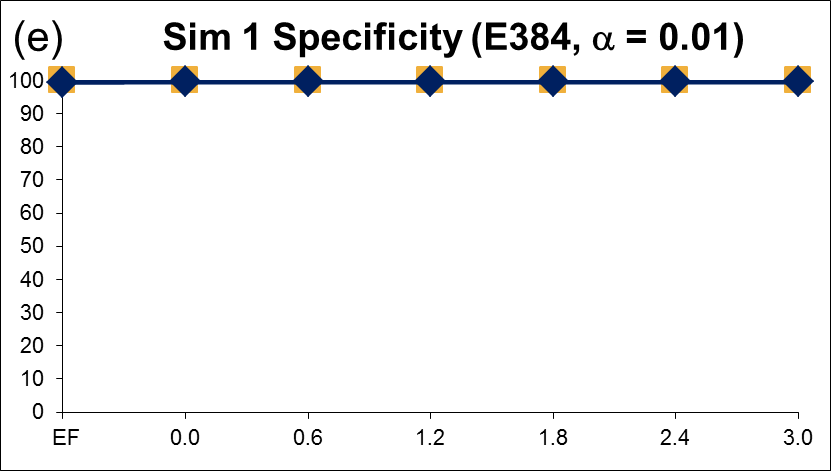

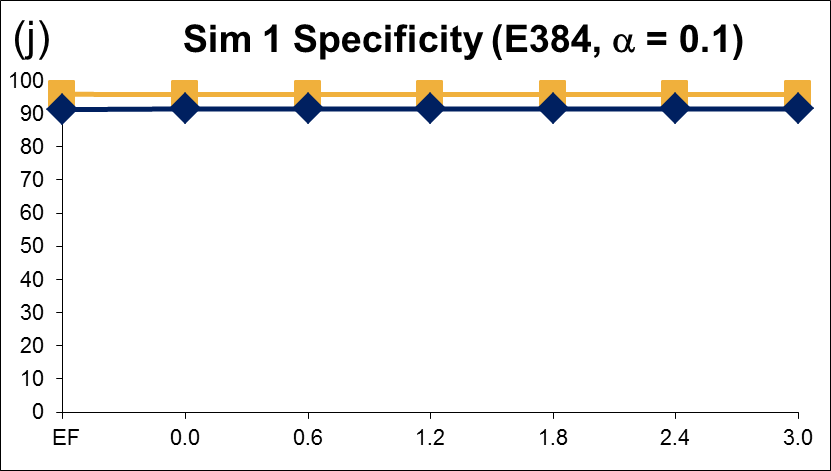


## Figure 6SM - Simulation 1, Plate Size: 1536 wells – Specificity (True Negative Rate)

Systematic error size: 10% (at most 8 columns and 8 rows affected). First column: cases (a) - (e): *α* = 0.01; Second column: cases (f) - (j): *α* = 0.1. Systematic Error Detection Tests: (⧫) t-test and (◼) K-S test.


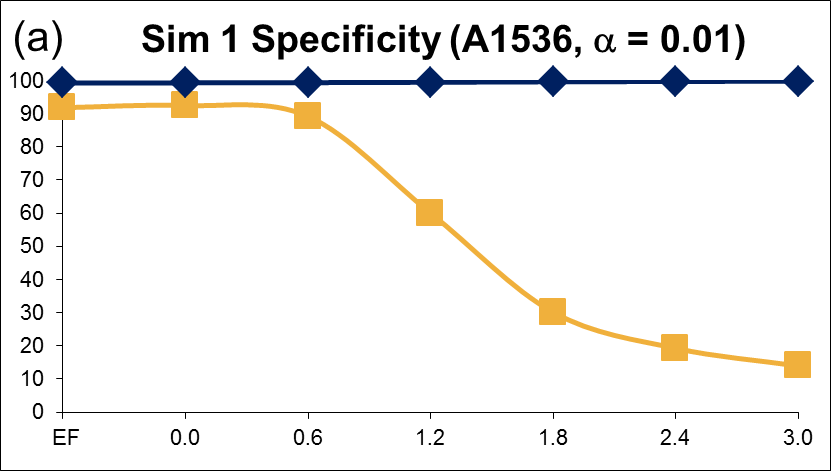

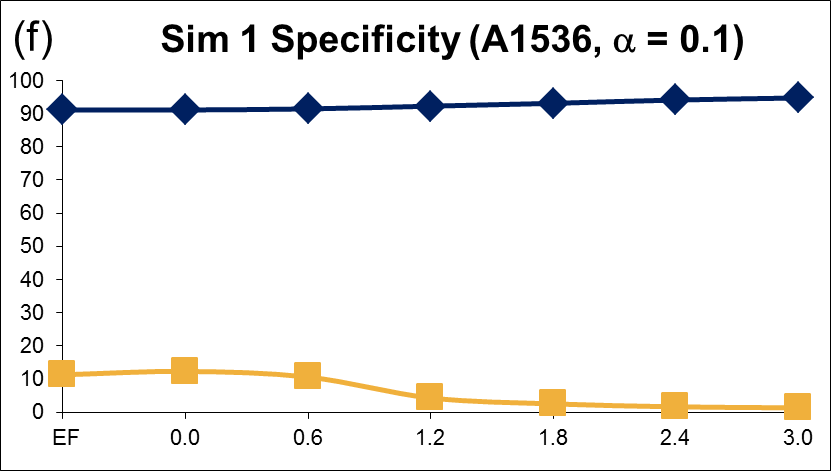


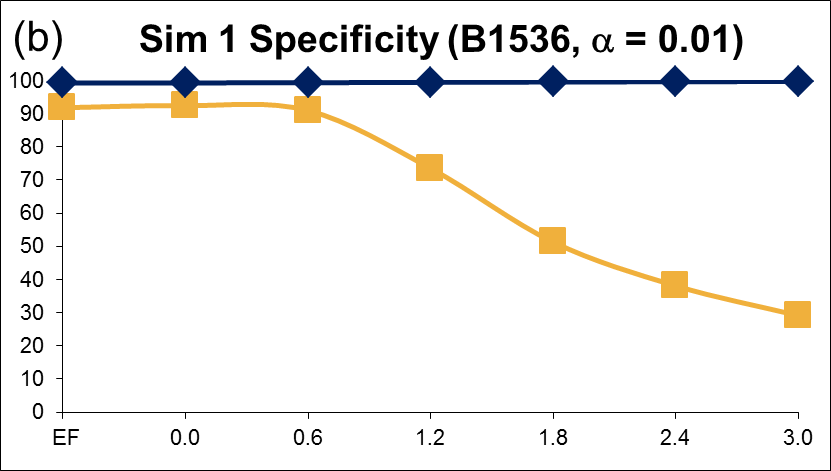

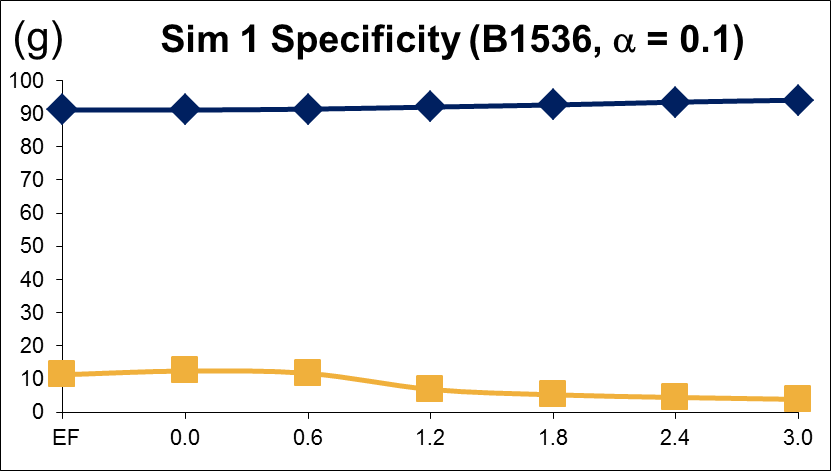


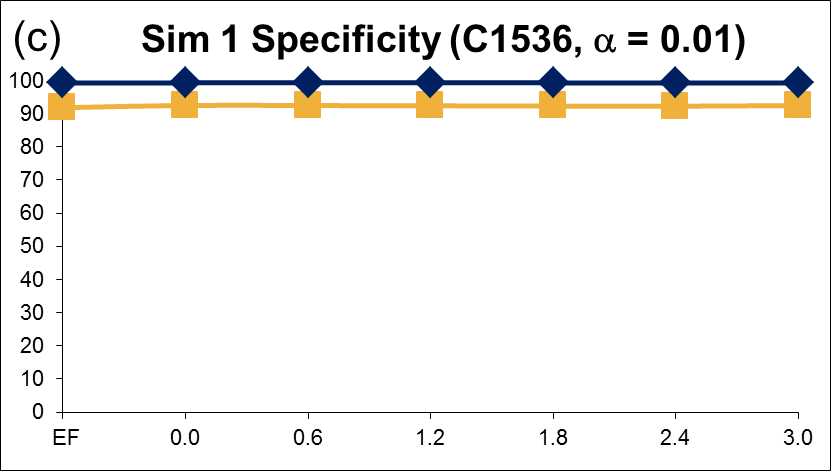

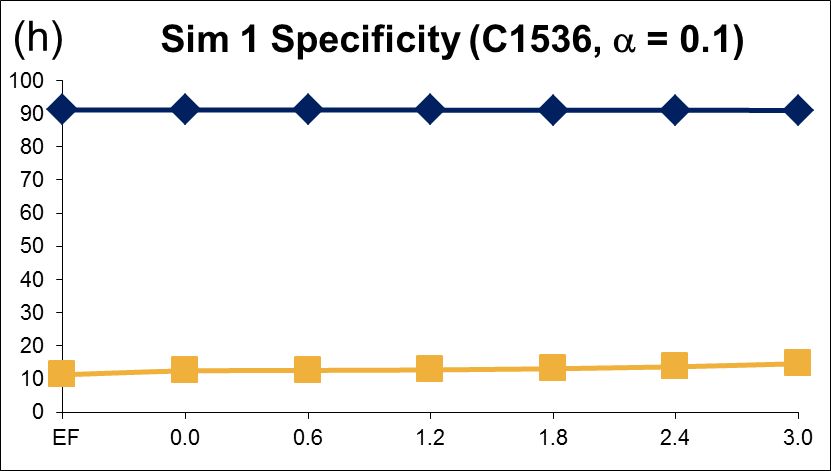


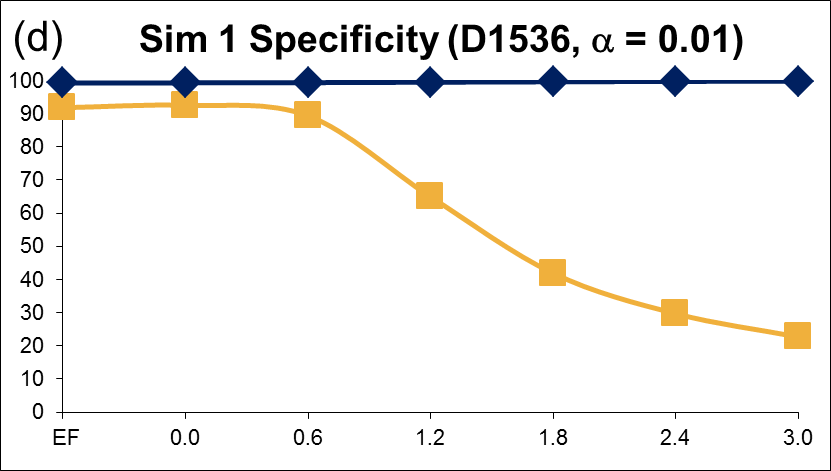

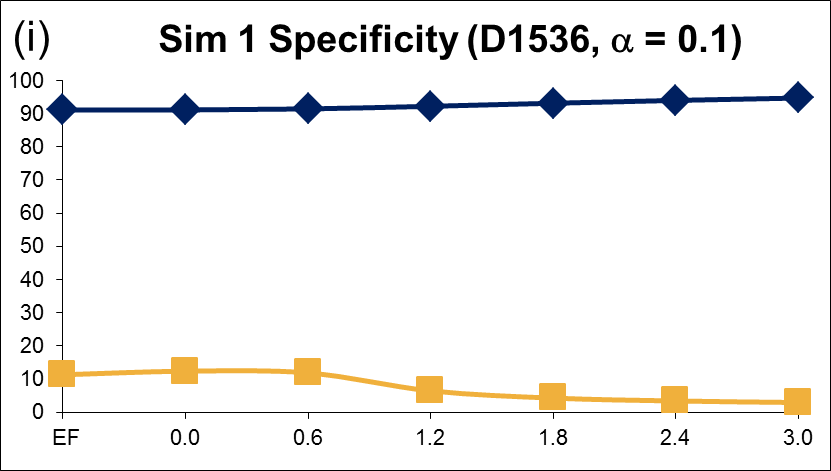


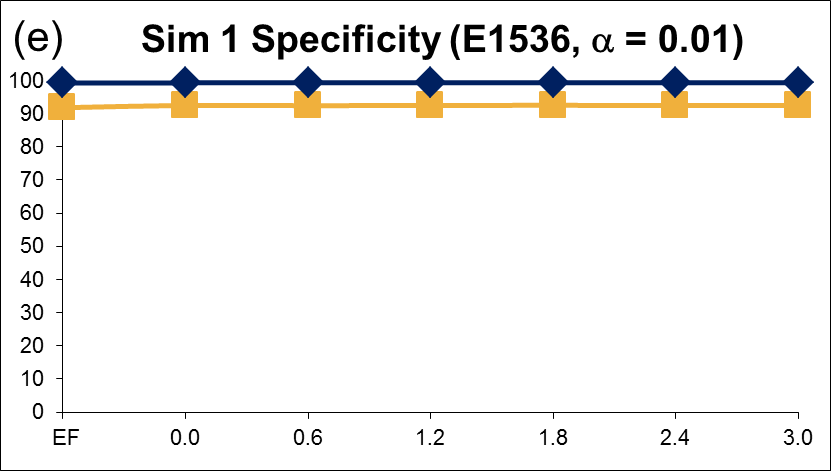

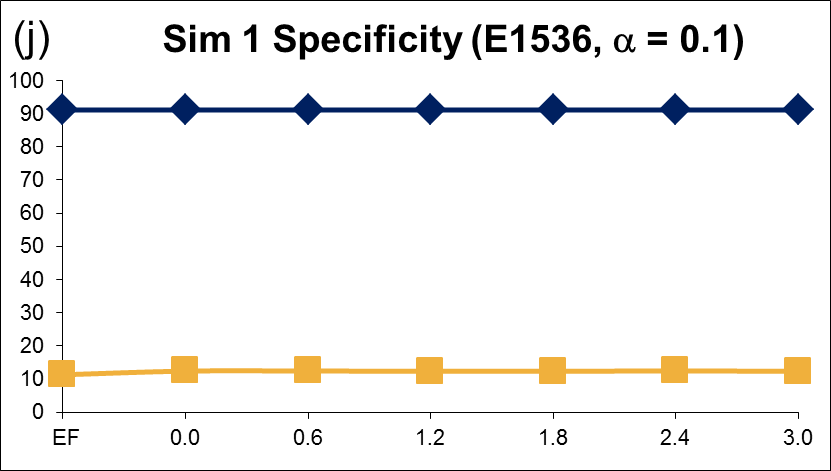


## Figure 7SM - Simulation 2, Plate Size: 96 wells - Sensitivity (True Positive Rate)

Systematic error size: 10% (at most 2 columns and 2 rows affected). First column: cases (a) - (b): *α* = 0.01; Second column: cases (c) - (d): *α* = 0.1. Systematic Error Detection Tests: (⧫) t-test, (◼) K-S test and (▲)$\chi^{2}$ goodness-of-fit test.


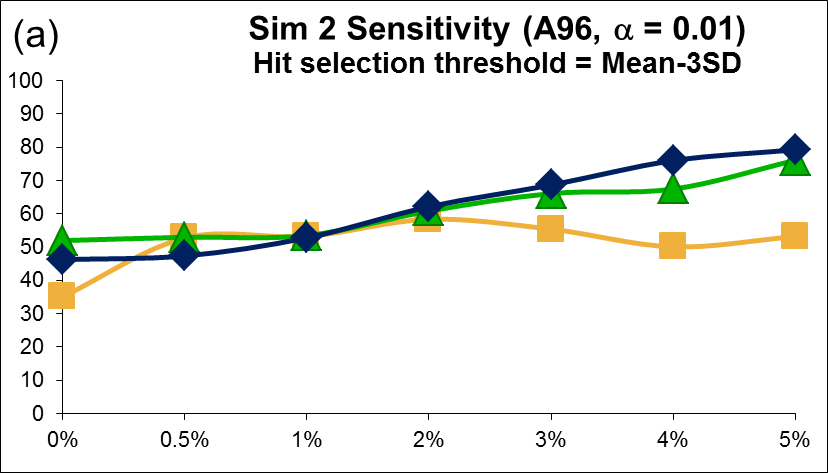

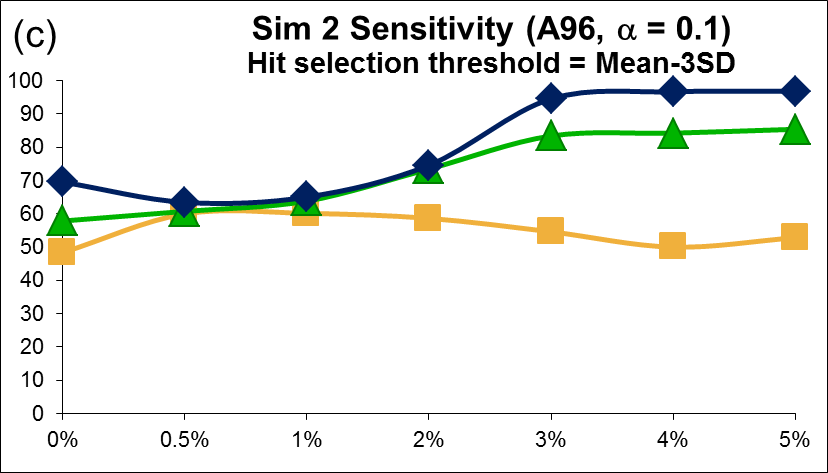


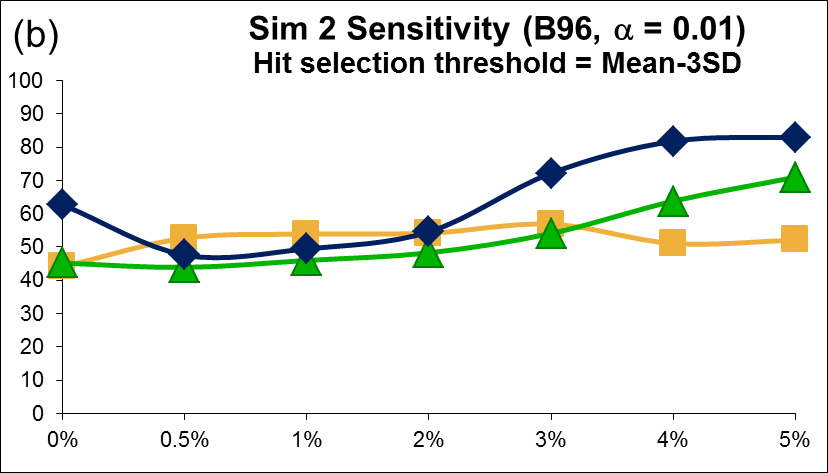

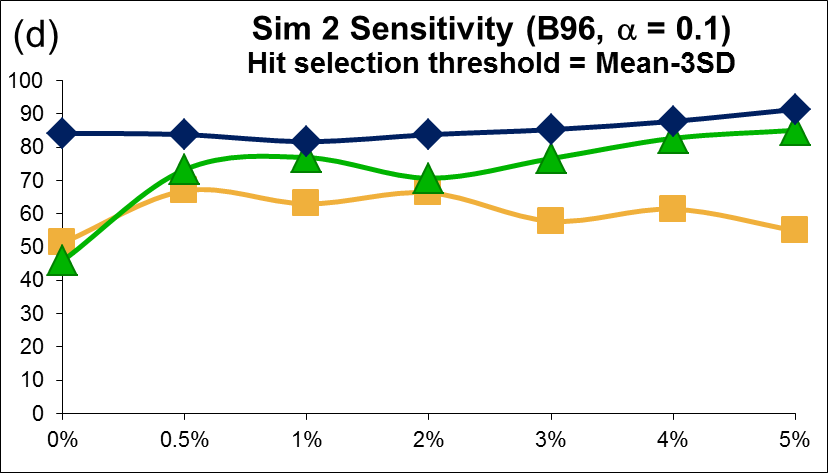


## Figure 8SM - Simulation 2, Plate Size: 384 wells - Sensitivity (True Positive Rate)

Systematic error size: 10% (at most 4 columns and 4 rows affected). First column: cases (a) - (b): *α* = 0.01; Second column: cases (c) - (d): *α* = 0.1. Systematic Error Detection Tests: (⧫) t-test, (◼) K-S test and (▲)$\chi^{2}$ goodness-of-fit test.


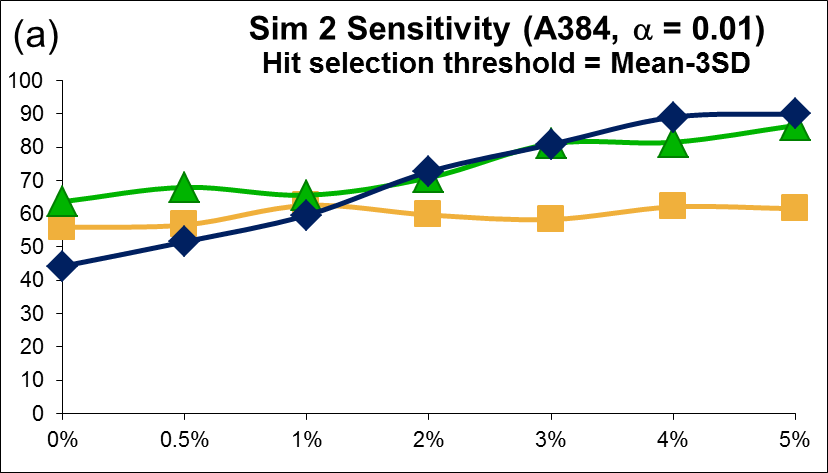

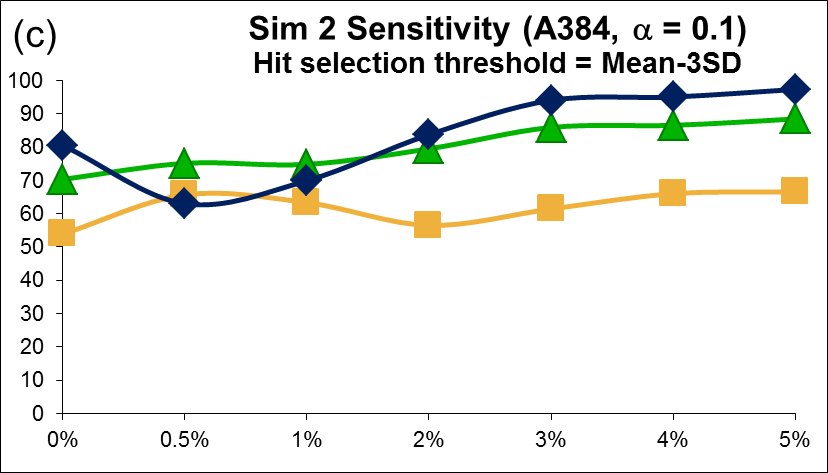


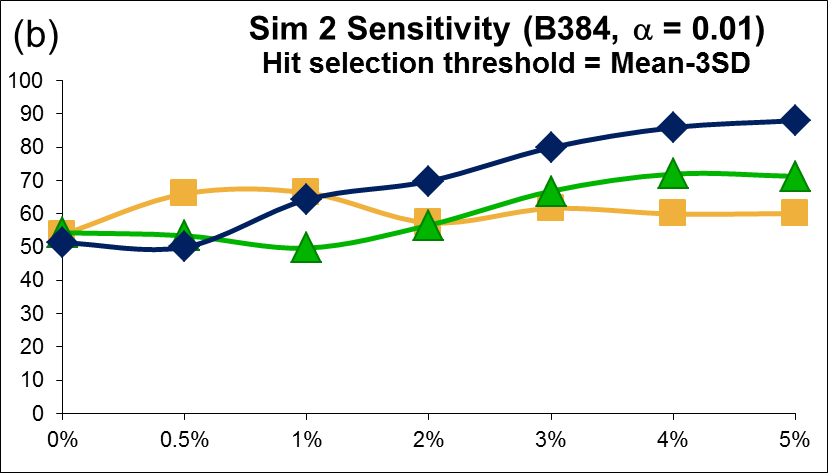

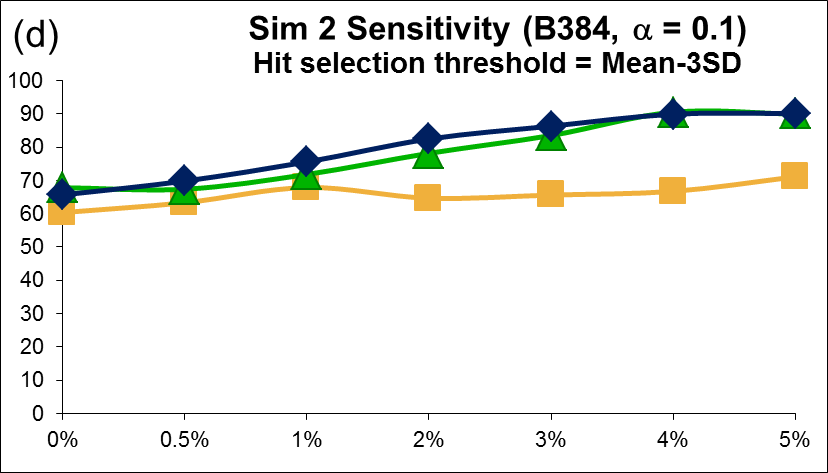


## Figure 9SM - Simulation 2, Plate Size: 1536 wells - Sensitivity (True Positive Rate)

Systematic error size: 10% (at most 8 columns and 8 rows affected). First column: cases (a) - (b): *α* = 0.01; Second column: cases (c) - (d): *α* = 0.1. Systematic Error Detection Tests: (⧫) t-test, (◼) K-S test and (▲)$\chi^{2}$ goodness-of-fit test.


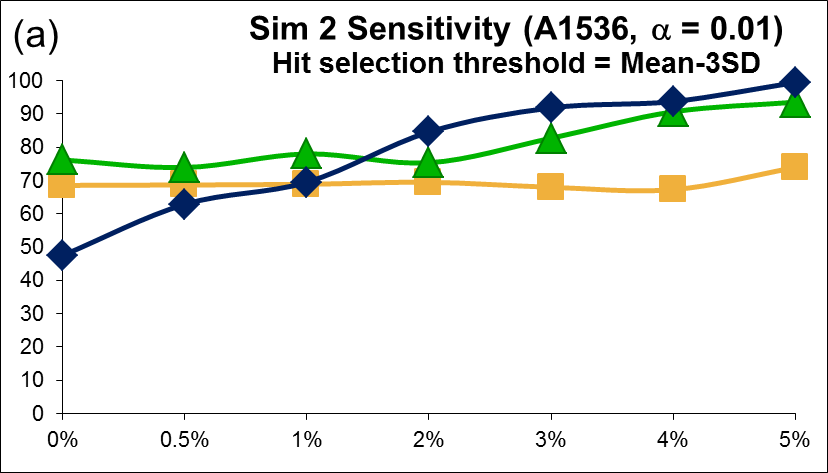

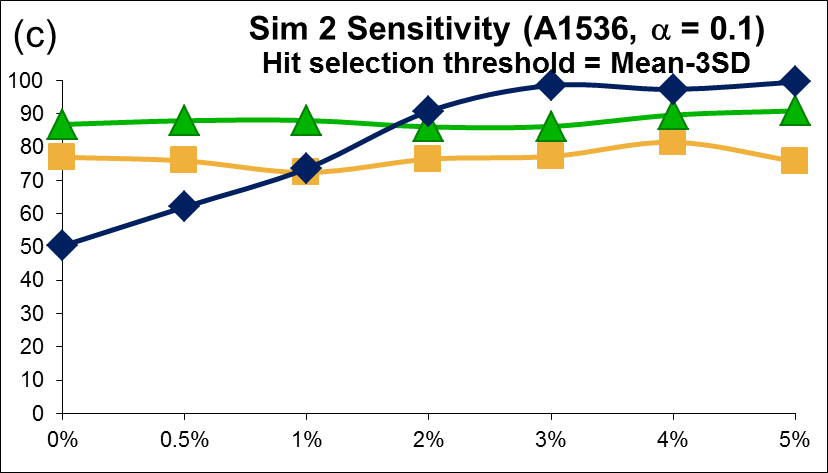


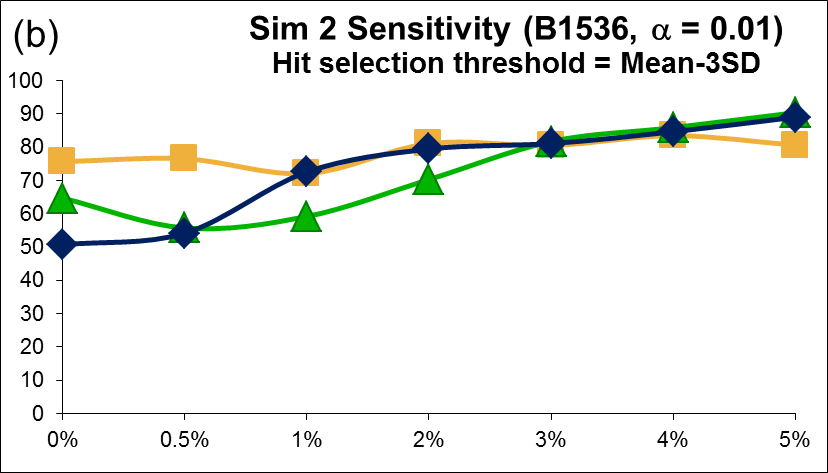

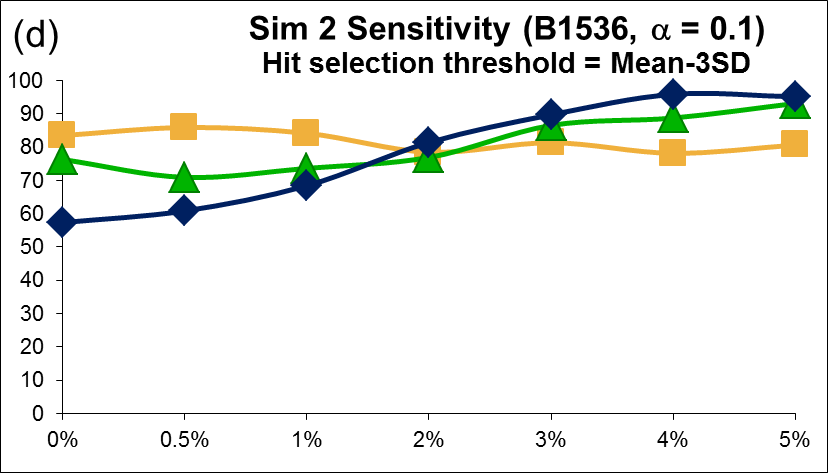


## Figure 10SM - Simulation 2, Plate Size: 96 wells - Specificity (True Negative Rate)

Systematic error size: 10% (at most 2 columns and 2 rows affected). First column: cases (a) - (e): *α* = 0.01; Second column: cases (f) - (j): *α* = 0.1. Systematic Error Detection Tests: (⧫) t-test, (◼) K-S test and (▲)$\chi^{2}$ goodness-of-fit test.


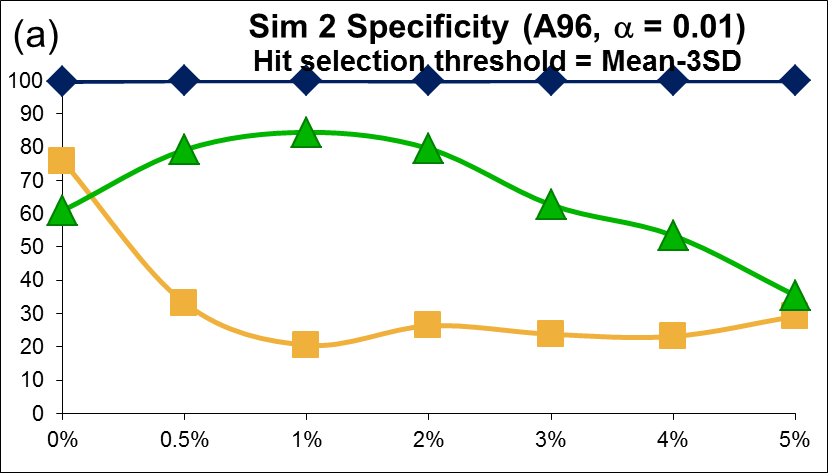

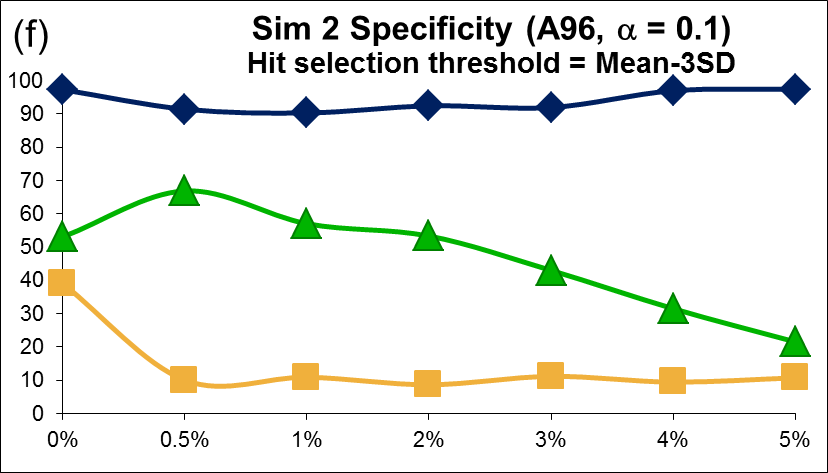


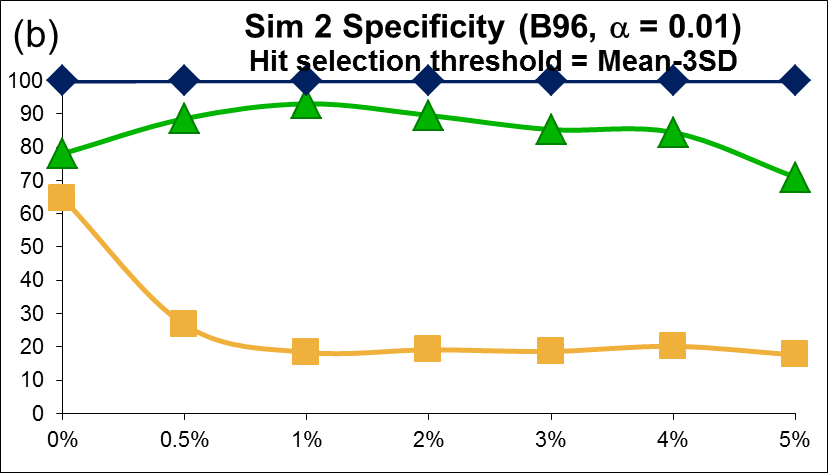

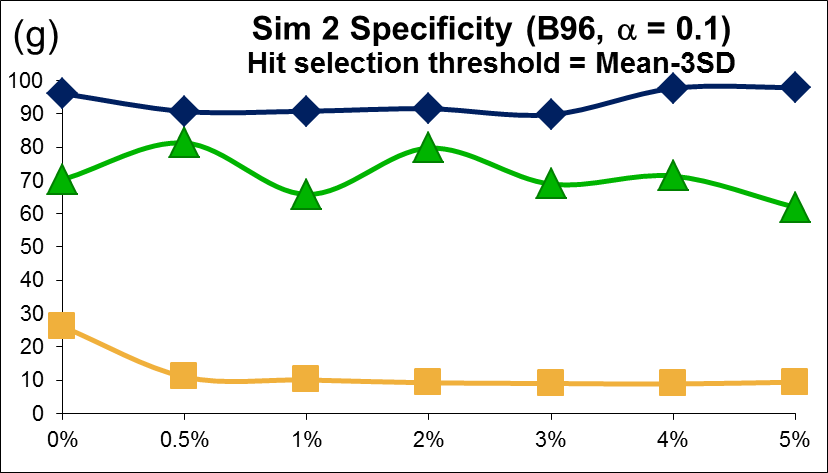


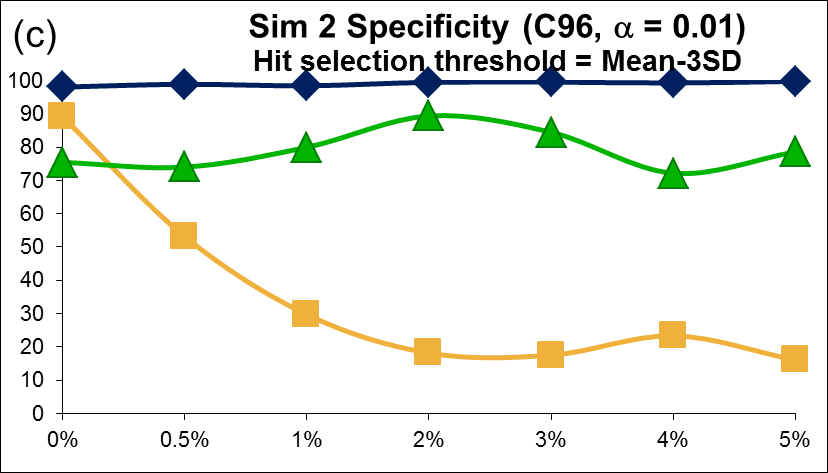

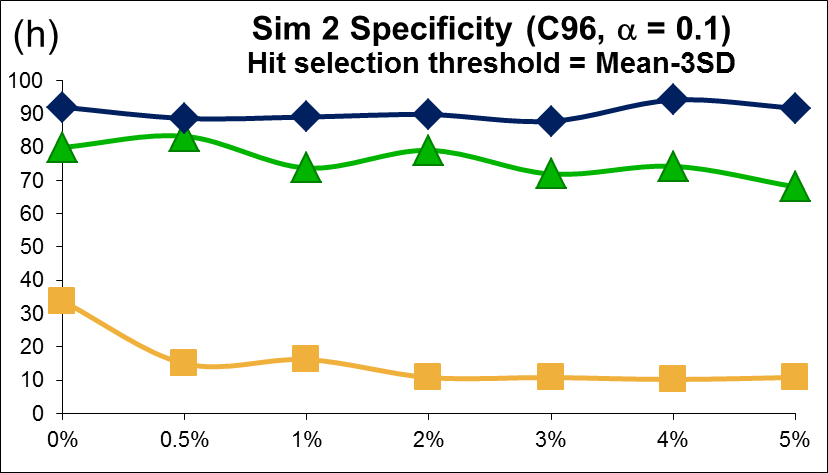


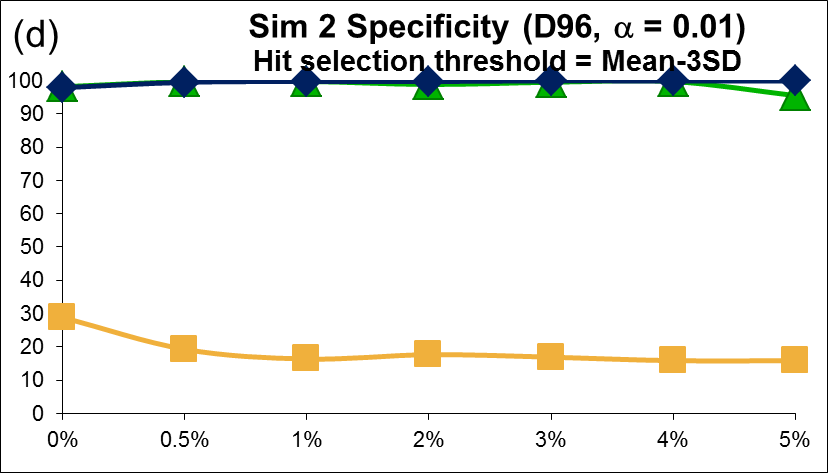

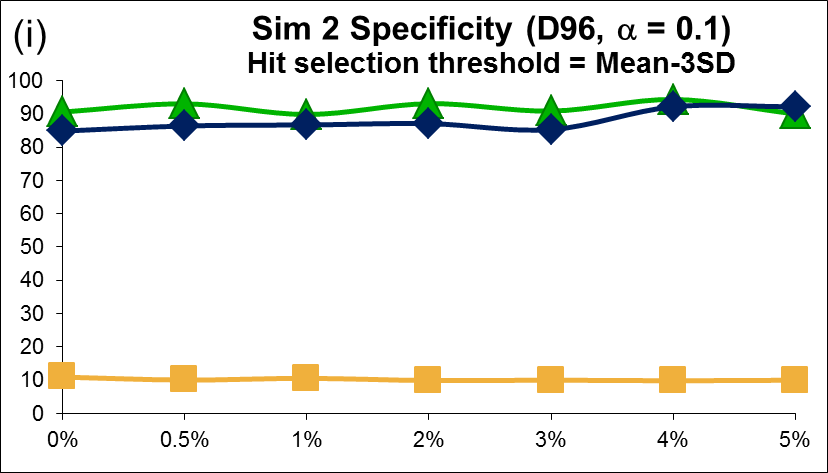


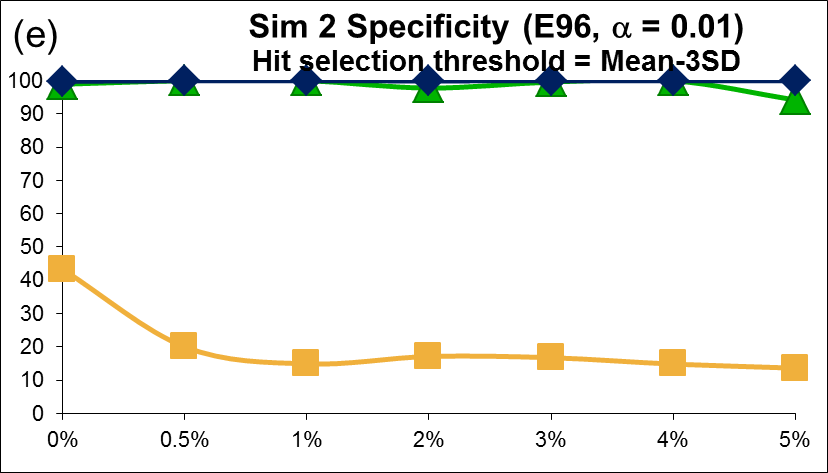

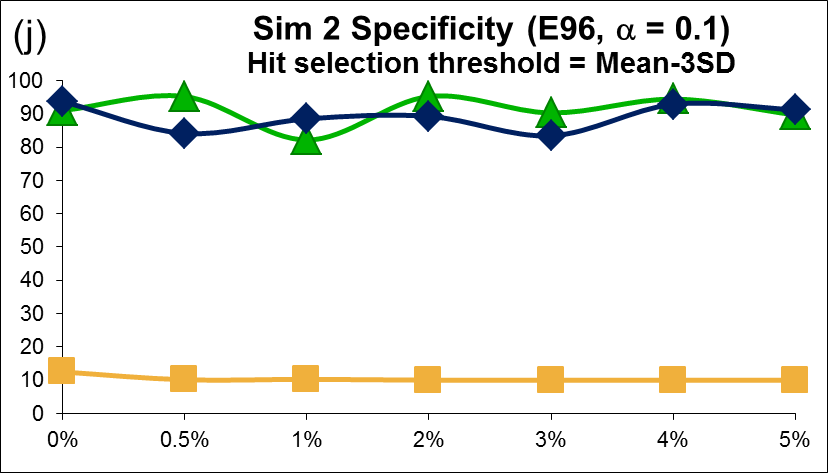


## Figure 11SM - Simulation 2, Plate Size: 384 wells - Specificity (True Negative Rate)

Systematic error size: 10% (at most 4 columns and 4 rows affected). First column: cases (a) - (e): *α* = 0.01; Second column: cases (f) - (j): *α* = 0.1. Systematic Error Detection Tests: (⧫) t-test, (◼) K-S test and (▲)$\chi^{2}$ goodness-of-fit test.


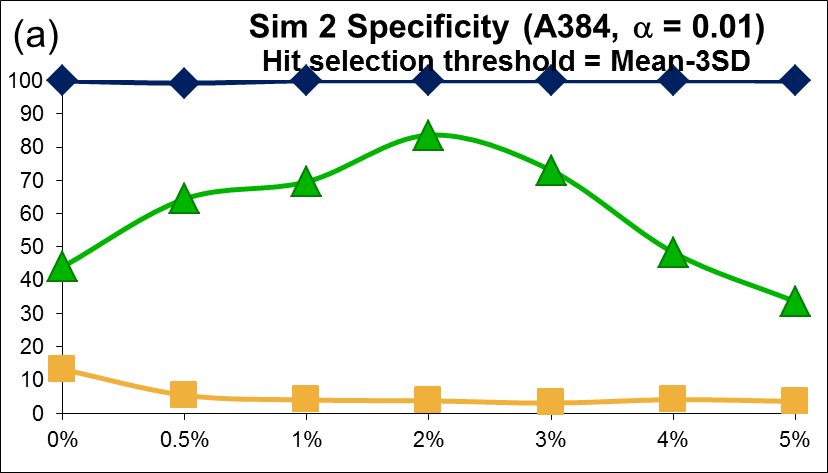

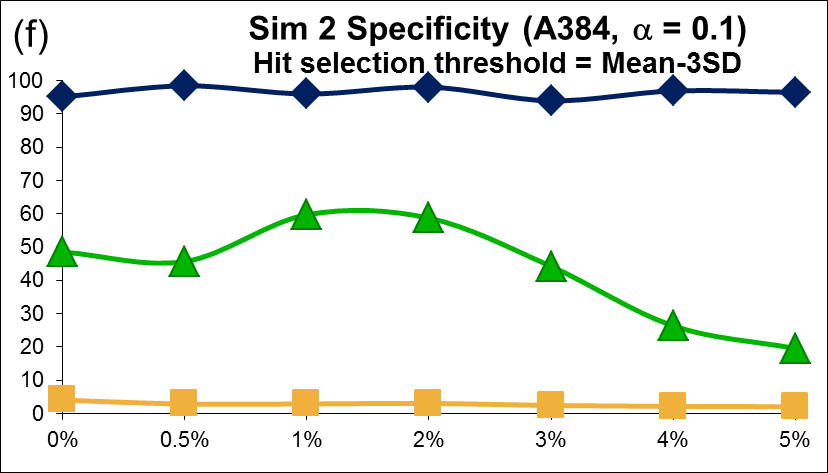


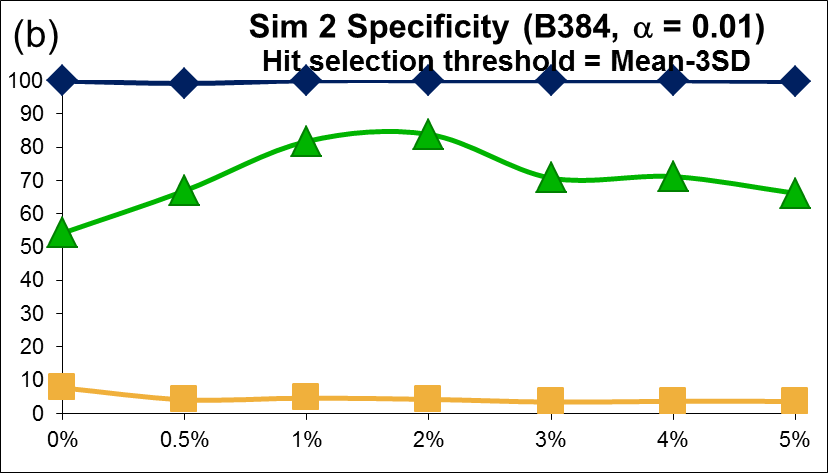

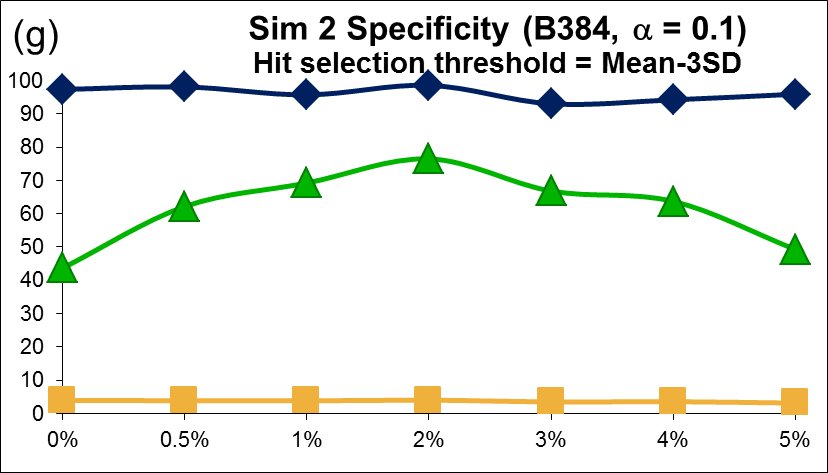


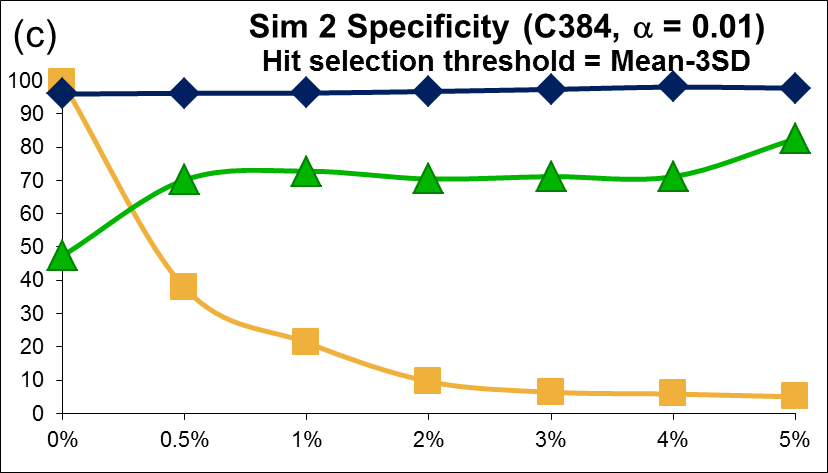

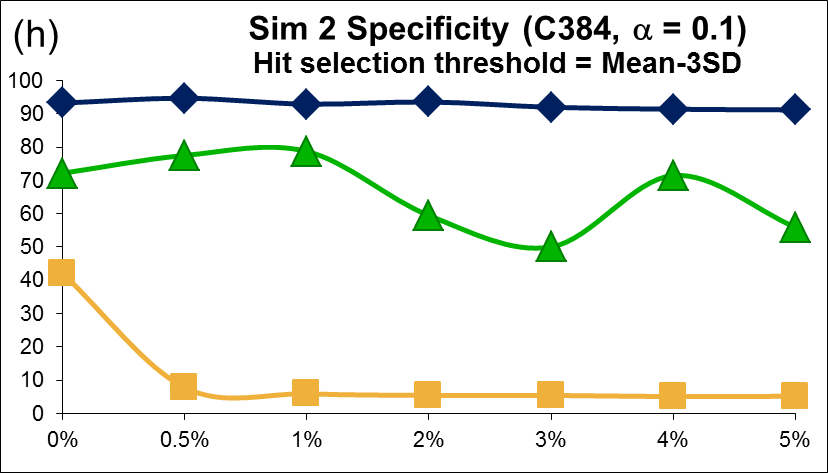


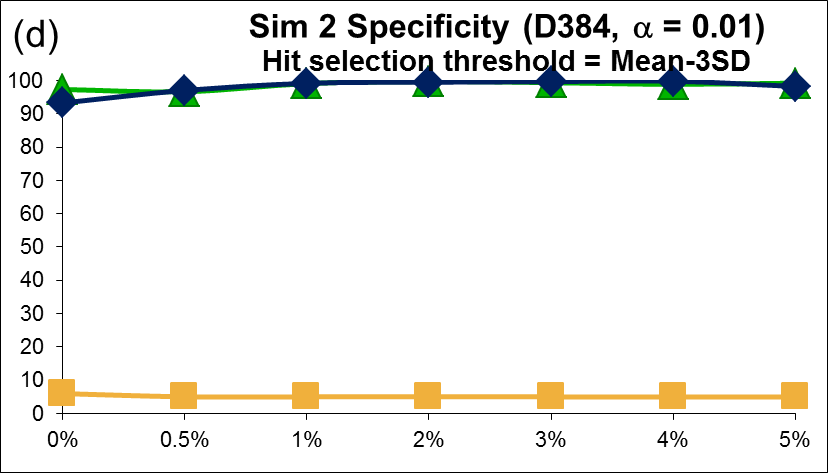

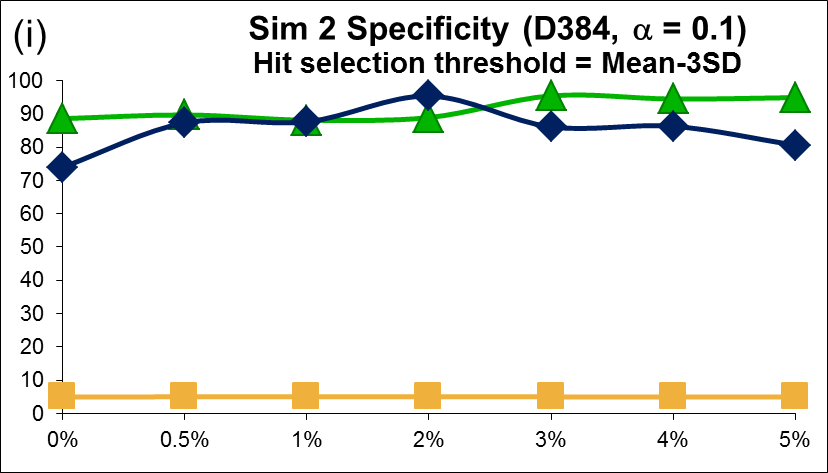


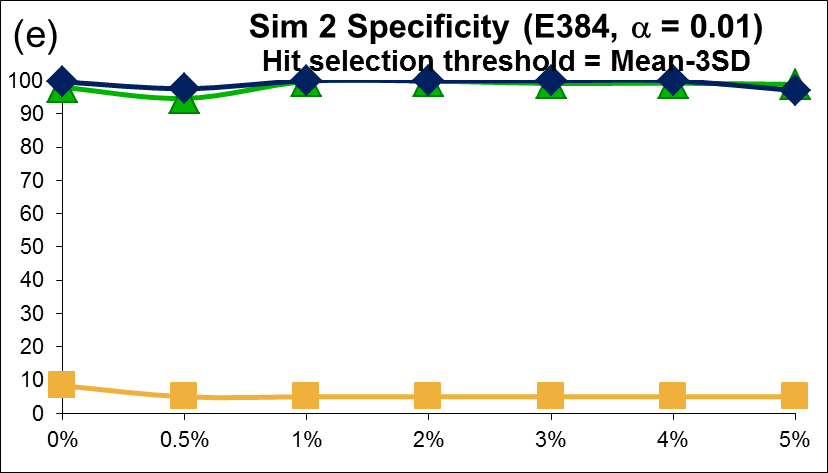

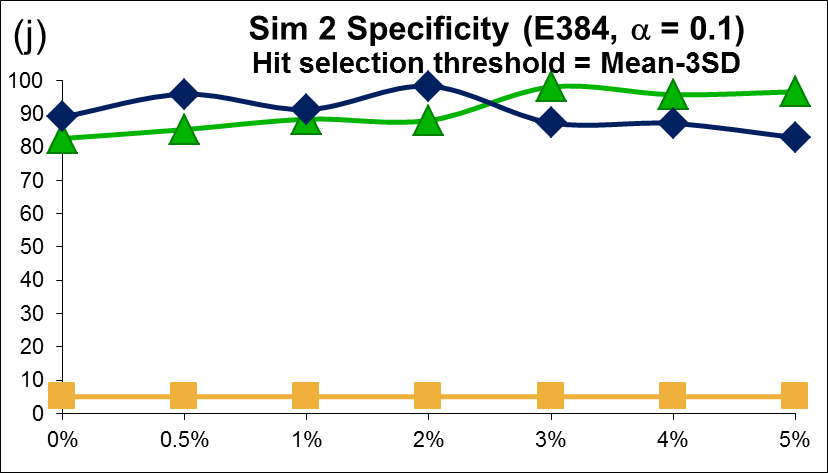


## Figure 12SM - Simulation 2, Plate Size: 1536 wells - Specificity (True Negative Rate)

Systematic error size: 10% (at most 8 columns and 8 rows affected). First column: cases (a) - (e): *α* = 0.01; Second column: cases (f) - (j): *α* = 0.1. Systematic Error Detection Tests: (⧫) t-test, (◼) K-S test and (▲)$\chi^{2}$ goodness-of-fit test.


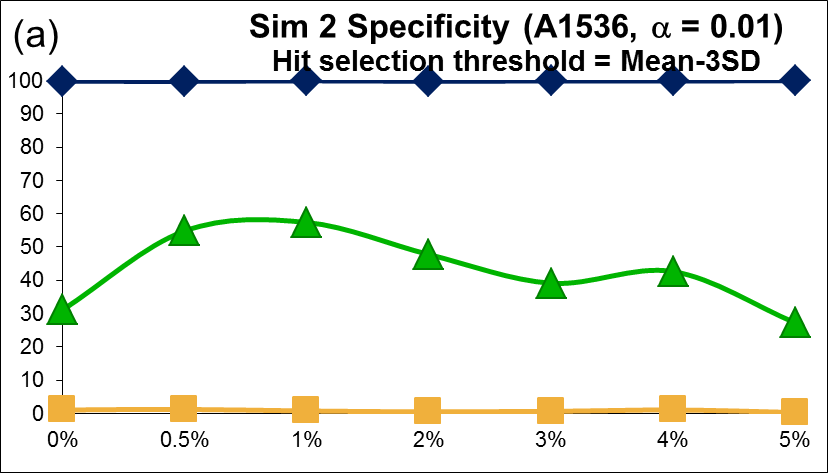

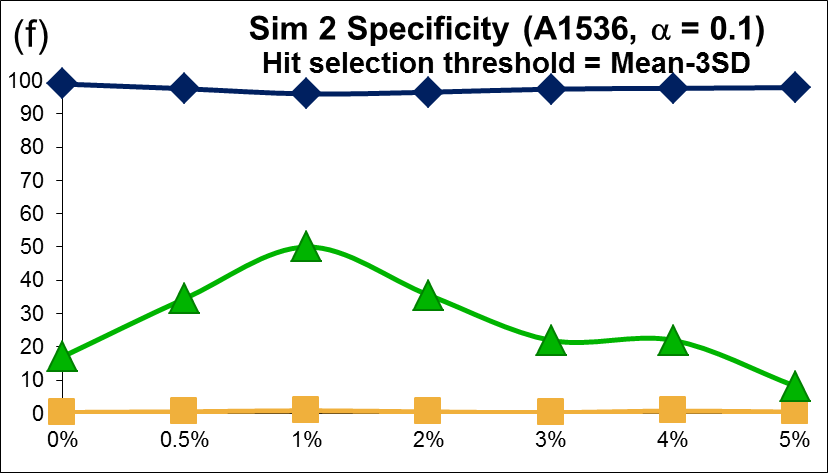


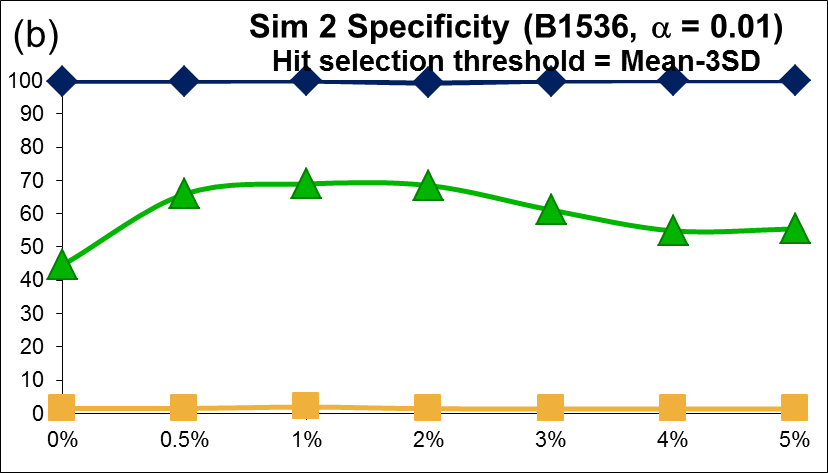

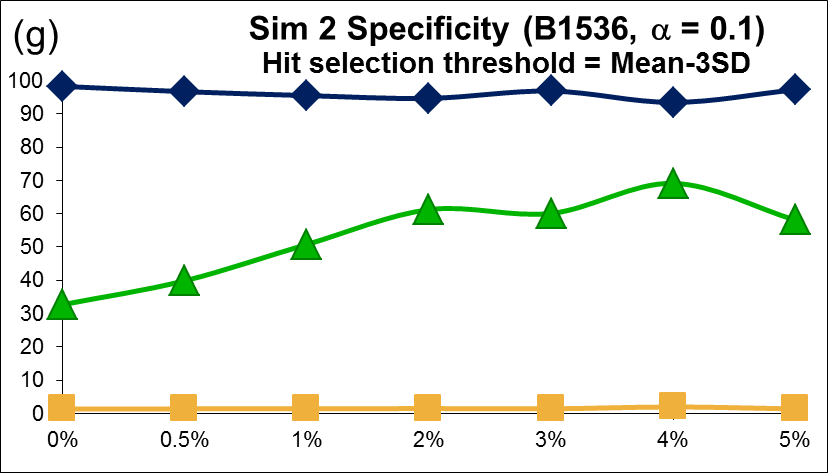


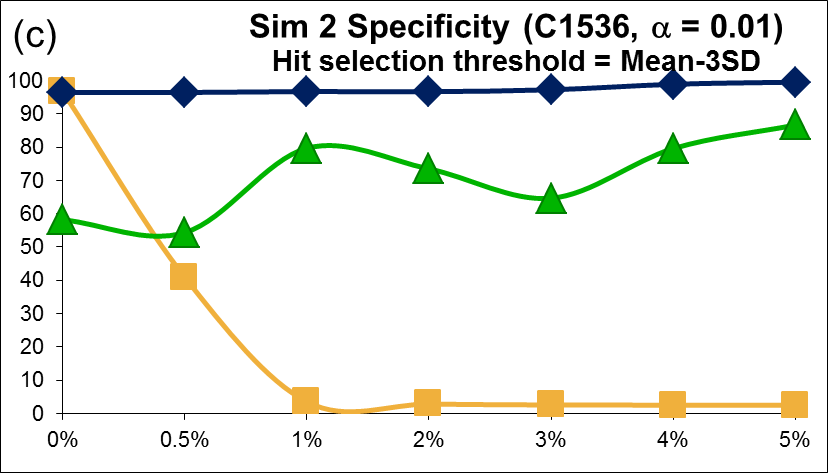

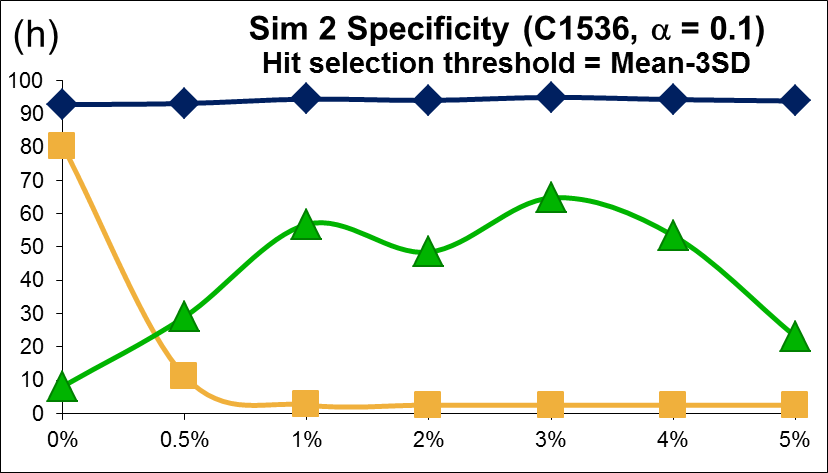


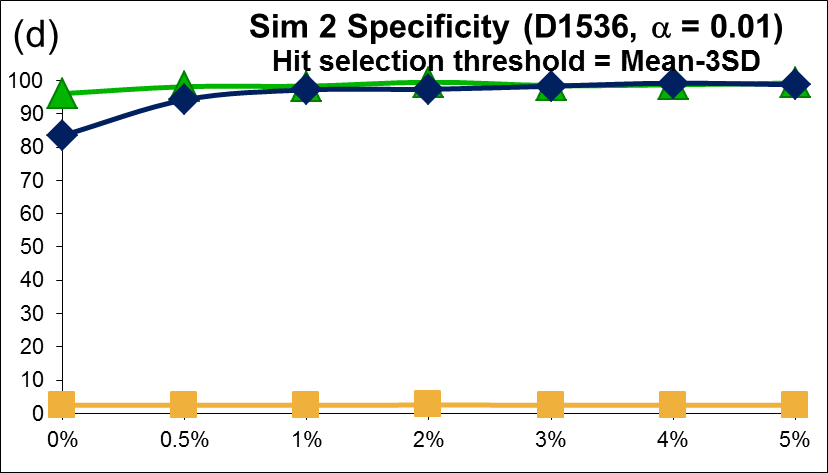

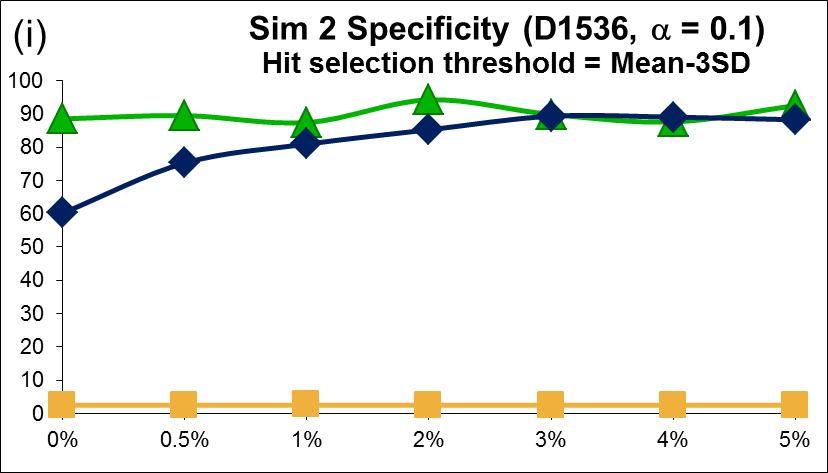


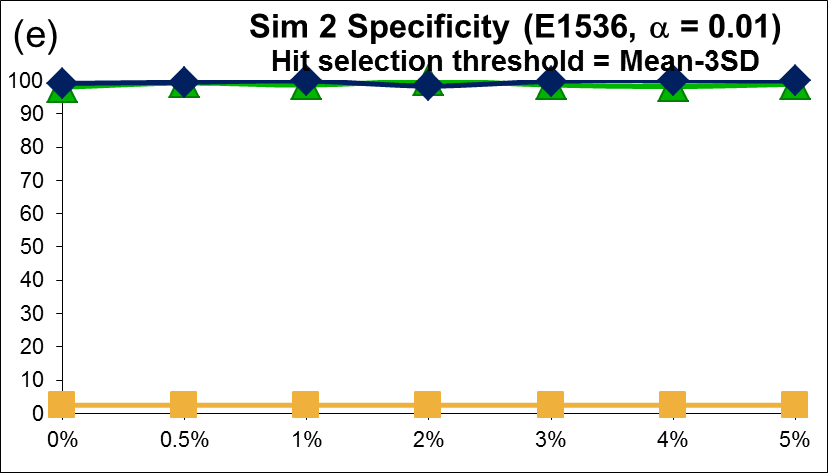

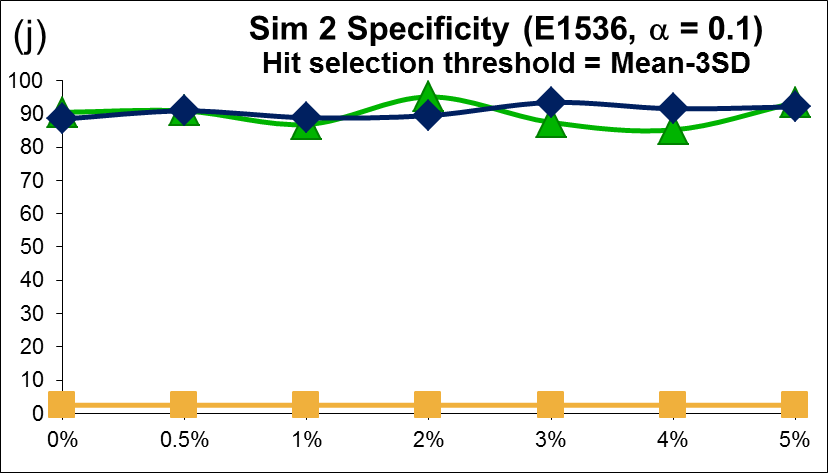


## Figure 13SM - Simulation 1, Plate Size: 96 wells – Success Rate

Systematic error size: 10% (at most 2 columns and 2 rows affected). First column: cases (a) - (e): *α* = 0.01; Second column: cases (f) - (j): *α* = 0.1. Systematic Error Detection Tests: (⧫) t-test and (◼) K-S test.


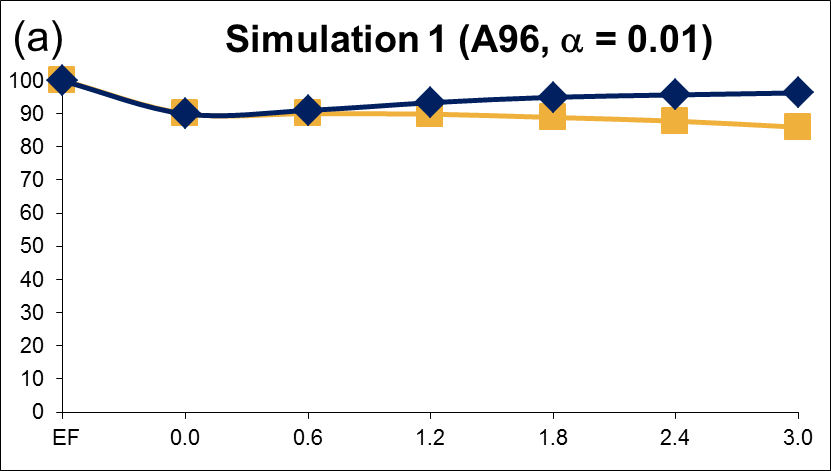

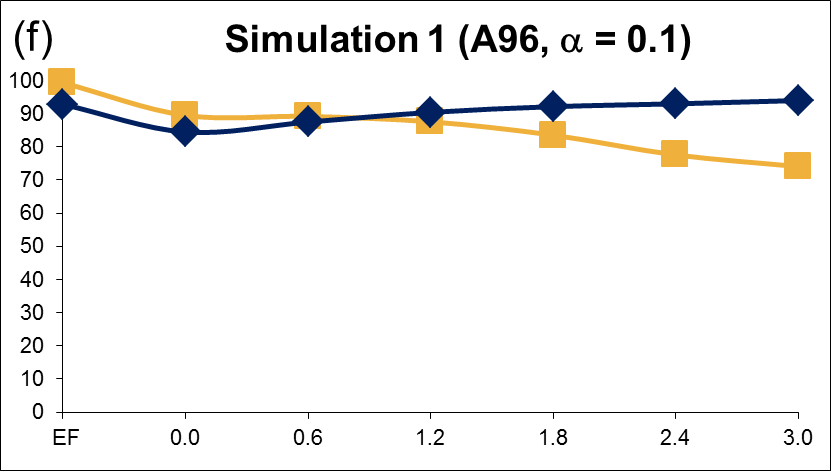


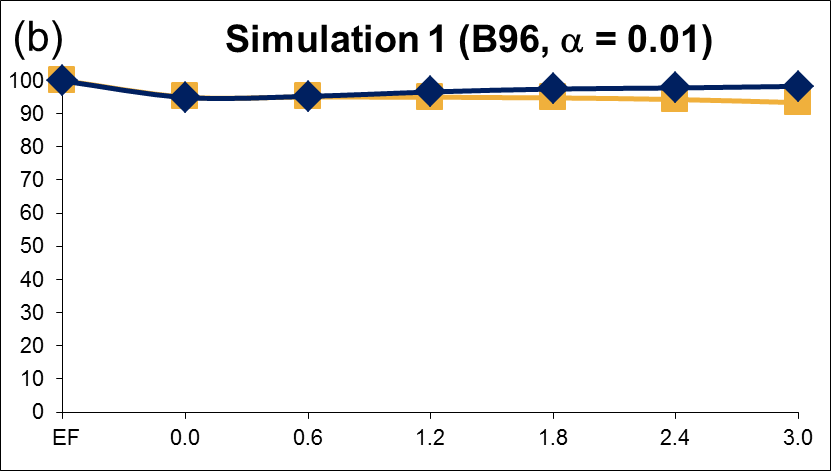

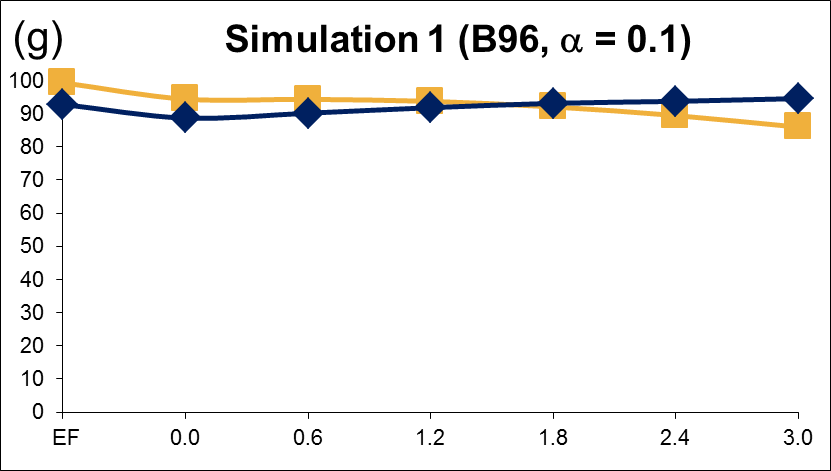


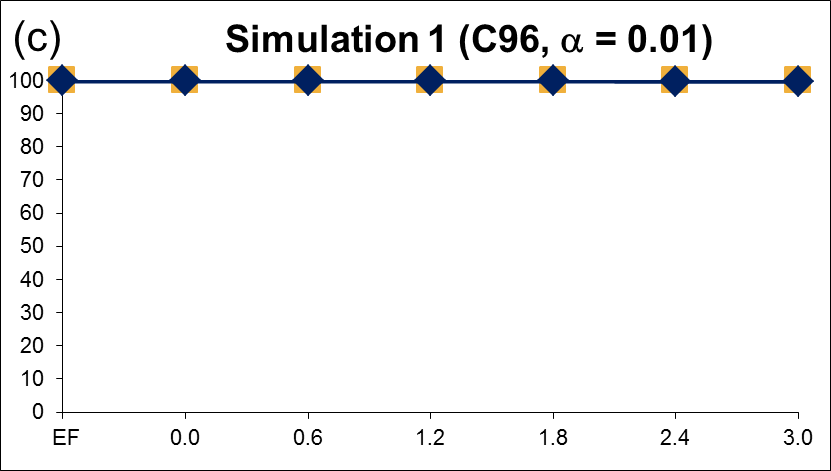

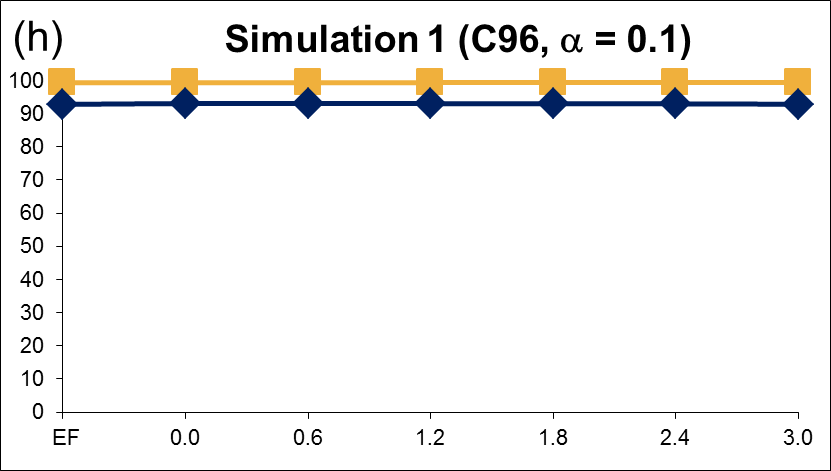


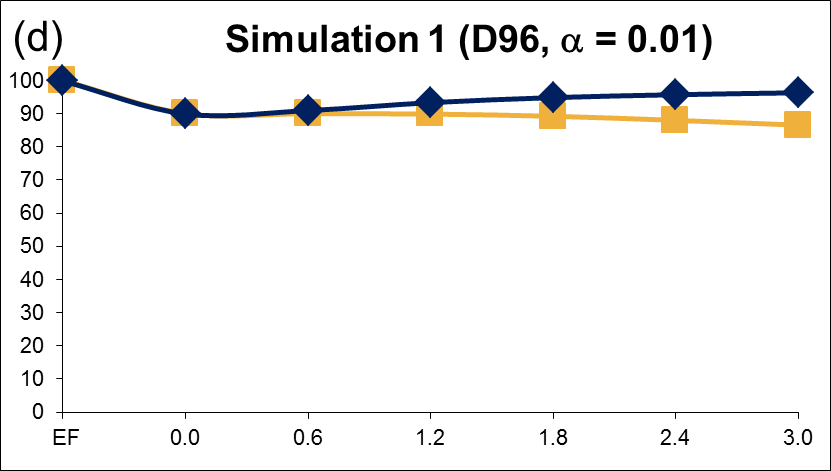

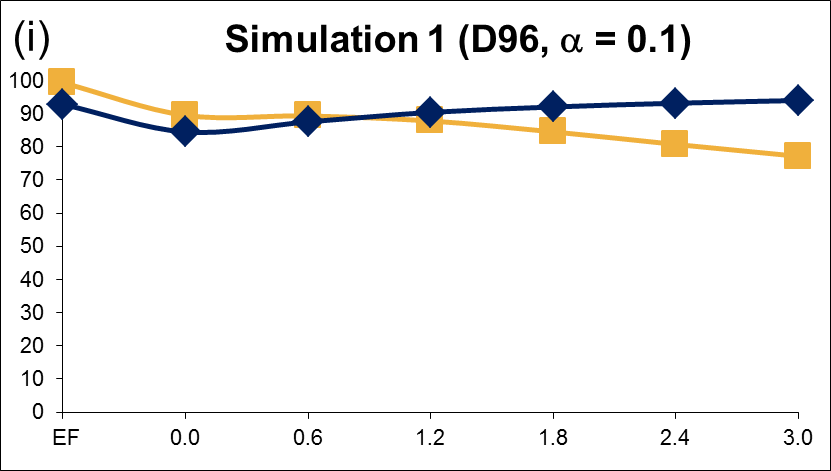


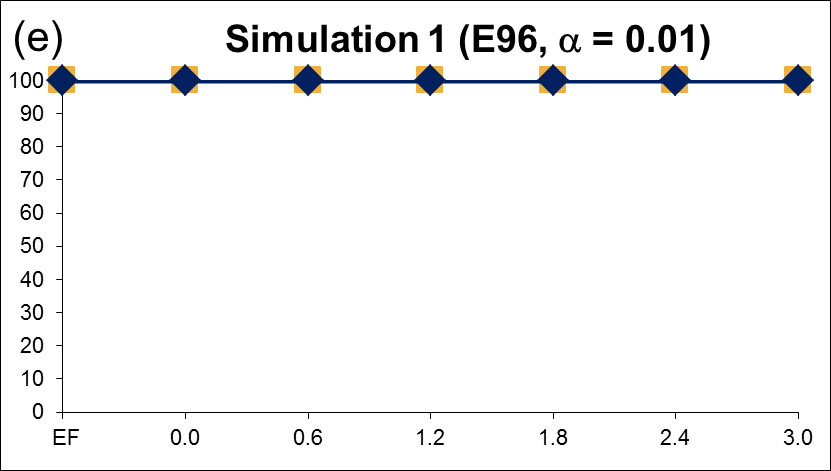

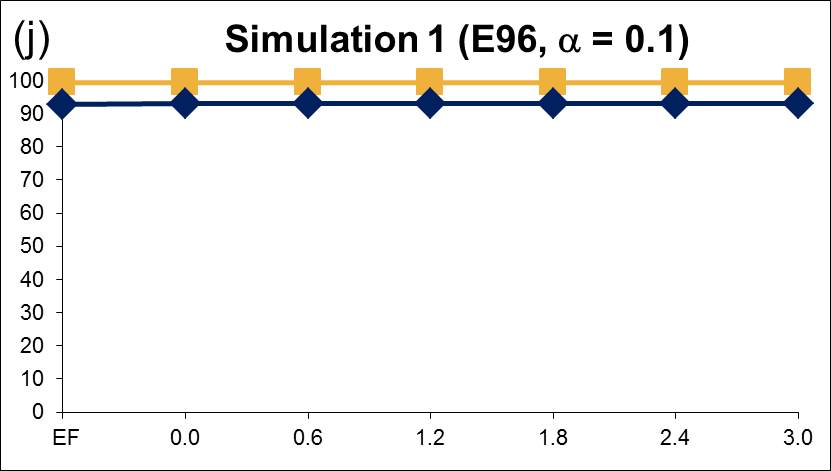


## Figure 14SM - Simulation 1, Plate Size: 384 wells – Success Rate

Systematic error size: 10% (at most 4 columns and 4 rows affected). First column: cases (a) - (e): *α* = 0.01; Second column: cases (f) - (j): *α* = 0.1. Systematic Error Detection Tests: (⧫) t-test and (◼) K-S test.

## Figure 15SM - Simulation 1, Plate Size: 1536 wells – Success Rate

Systematic error size: 10% (at most 8 columns and 8 rows affected). First column: cases (a) - (e): *α* = 0.01; Second column: cases (f) - (j): *α* = 0.1. Systematic Error Detection Tests: (⧫) t-test and (◼) K-S test.

## Figure 16SM - Simulation 2, Plate Size: 96 wells - Success Rate

Systematic error size: 10% (at most 2 columns and 2 rows affected). First column: cases (a) - (e): *α* = 0.01; Second column: cases (f) - (j): *α* = 0.1. Systematic Error Detection Tests: (⧫) t-test, (◼) K-S test and (▲)$\chi^{2}$ goodness-of-fit test.

## Figure 17SM - Simulation 2, Plate Size: 384 wells - Success Rate

Systematic error size: 10% (at most 4 columns and 4 rows affected). First column: cases (a) - (e): *α* = 0.01; Second column: cases (f) - (j): *α* = 0.1. Systematic Error Detection Tests: (⧫) t-test, (◼) K-S test and (▲)$\chi^{2}$ goodness-of-fit test.

## Figure 18SM - Simulation 2, Plate Size: 1536 wells - Success Rate

Systematic error size: 10% (at most 8 columns and 8 rows affected). First column: cases (a) - (e): *α* = 0.01; Second column: cases (f) - (j): *α* = 0.1. Systematic Error Detection Tests: (⧫) t-test, (◼) K-S test and (▲)$\chi^{2}$ goodness-of-fit test.

## Figure 19SM – Data distribution before and after the application of the Discrete Fourier Transform (DFT) method

Data from one of the simulated 96-well plates before and after the application of Discrete Fourier Transform. The raw data followed a normal distribution and contained ****random error only**** (i.e., systematic error was not added). The raw data show agreement with the normal distribution, both graphically (case a) and by the Kolmogorov-Smirnov test (*KS* = 0.03, *p* = 0.5). However, after the application of Discrete Fourier Transform, the data deviate from normality as shown in the graph (case b) and by the Kolmogorov-Smirnov test (*KS* = 0.06, *p* = 0.0018).

|  |  |
| --- | --- |

(a) (b)
